# A powerful method for pleiotropic analysis under composite null hypothesis identifies novel shared loci between Type 2 Diabetes and Prostate Cancer

**DOI:** 10.1371/journal.pgen.1009218

**Published:** 2020-12-08

**Authors:** Debashree Ray, Nilanjan Chatterjee

**Affiliations:** 1 Department of Epidemiology, Bloomberg School of Public Health, Johns Hopkins University, Baltimore, Maryland, United States of America; 2 Department of Biostatistics, Bloomberg School of Public Health, Johns Hopkins University, Baltimore, Maryland, United States of America; 3 Department of Oncology, School of Medicine, Johns Hopkins University, Baltimore, Maryland, United States of America; Emory University, UNITED STATES

## Abstract

There is increasing evidence that pleiotropy, the association of multiple traits with the same genetic variants/loci, is a very common phenomenon. Cross-phenotype association tests are often used to jointly analyze multiple traits from a genome-wide association study (GWAS). The underlying methods, however, are often designed to test the global null hypothesis that there is no association of a genetic variant with any of the traits, the rejection of which does not implicate pleiotropy. In this article, we propose a new statistical approach, PLACO, for specifically detecting pleiotropic loci between two traits by considering an underlying composite null hypothesis that a variant is associated with none or only one of the traits. We propose testing the null hypothesis based on the product of the Z-statistics of the genetic variants across two studies and derive a null distribution of the test statistic in the form of a mixture distribution that allows for fractions of variants to be associated with none or only one of the traits. We borrow approaches from the statistical literature on mediation analysis that allow asymptotic approximation of the null distribution avoiding estimation of nuisance parameters related to mixture proportions and variance components. Simulation studies demonstrate that the proposed method can maintain type I error and can achieve major power gain over alternative simpler methods that are typically used for testing pleiotropy. PLACO allows correlation in summary statistics between studies that may arise due to sharing of controls between disease traits. Application of PLACO to publicly available summary data from two large case-control GWAS of Type 2 Diabetes and of Prostate Cancer implicated a number of novel shared genetic regions: 3q23 (*ZBTB38*), 6q25.3 (*RGS17*), 9p22.1 (*HAUS6*), 9p13.3 (*UBAP2*), 11p11.2 (*RAPSN*), 14q12 (*AKAP6*), 15q15 (*KNL1*) and 18q23 (*ZNF236*).

## Author summary

We propose a new approach PLACO that uses aggregate-level genotype-phenotype association statistics—commonly referred to as GWAS summary statistics—to identify genetic variants that influence risk of two traits or diseases. It allows correlation in summary statistics between studies that may arise due to sharing of controls between disease traits. We demonstrate that PLACO can achieve major power gain over alternative methods that are typically used. We applied PLACO to Type 2 Diabetes and Prostate Cancer summary data from two large case-control studies. Many previous studies have reported an inverse association of these two chronic diseases suggesting shared risk factors; however, shared genetic mechanisms underlying this association is poorly understood. PLACO identified a number of novel shared genetic regions that are not detected by individual trait analysis. Many of the loci implicated by PLACO increase risk for one disease while decreasing risk for the other. PLACO can similarly be used on other traits to shed light on shared genetic risk factors.

## Introduction

Years of genetic research on various complex human traits have implicated numerous genetic variants as risk factors for two or more traits. Pleiotropy, the phenomenon where a genetic region or locus confers risk to more than one trait [1], is widely observed for many diseases and traits [2], especially cancers [3], autoimmune [4] and psychiatric [5, 6] disorders. It has also been observed in seemingly unrelated traits; for instance, early-onset androgenetic alopecia and Parkinson’s disease [7], Crohn’s disease and Parkinson’s disease [8], and coronary artery disease and tonsillectomy [9]. Pleiotropy provides new opportunities, as well as challenges, for diagnosis, therapeutics, and intervention on diseases [1, 2, 10, 11]. Consequently, it is important to identify and study shared genetic basis of complex traits.

To detect potential pleiotropic effects of genetic variants, many statistical methods for jointly analyzing multiple traits in genome-wide association studies (GWAS) have been proposed [1, 12, 13]. Use of these methods—commonly referred to as “cross-phenotype association tests”—has been gaining traction over the past few years, and has led to successful discovery and replication of genetic overlap among different human disorders and traits [5, 14–21]. Typical cross-phenotype association methods test the global null hypothesis that no trait is associated with a given genetic variant against the alternative hypothesis that at least one of the traits is associated. Thus, rejection of the null hypothesis could just be due to one trait being associated with the genetic variant, and not necessarily due to pleiotropy.

A number of Bayesian approaches exist that allow evaluation of pleiotropy on a genome-wide scale based on posterior probability of simultaneous association of a variant with two or more traits given GWAS summary data for each trait [12]. However, the power of these methods for detecting variant-level pleiotropy at specified family-wise error rate (FWER) or type I error rate are not well understood. For instance, conditional false discovery rate (FDR) approach [22], GPA [23] and their generalizations [24, 25] provide association mapping for a fixed FDR, which, unlike FWER, is more liberal and is not the standard GWAS error measure. Additionally, due to the higher level of complexity of Bayesian approaches and the well-established standard interpretations of frequentist approaches in GWAS, frequentist approaches are sometimes more appealing to researchers for association mapping.

In the frequentist realm, recently a few methods have been proposed to specifically test for pleiotropy, where the rejection of the null hypothesis of no pleiotropy is driven by the significant association of a genetic variant with more than one trait [26–29]. All of these methods require individual-level phenotype and genotype data on the same set of randomly sampled individuals, and cannot be readily extended to diseases on which case-control samples are available. While one may compare the significant variants of one trait with those of another, it is worth noting that the discovery of the variants in the first place may be under-powered in individual GWAS. Two other common strategies for examining genetic overlap between traits involve estimating genetic correlation, and testing how well polygenic risk score of one disease explains variation of the other. Both these approaches describe an overall genetic sharing, and do not indicate genetic sharing at a locus level or implicate novel shared variants/loci. To our knowledge, there is currently no summary statistics based frequentist approach to specifically test for pleiotropy between any two traits. Furthermore, there is no frequentist method for identifying pleiotropic loci between case-control traits that may or may not share controls.

In this article, we propose a formal statistical test of pleiotropy of two traits borrowing ideas from statistical mediation analysis literature. The proposed method, PLACO (**pl**eiotropic **a**nalysis under **co**mposite null hypothesis), can be applied to summary-level data available from GWAS of two traits and can account for potential correlation across traits, such as that arising due to shared controls in case-control studies. We conduct extensive simulation experiments to study type I error and power of PLACO at stringent significance levels. We apply PLACO to summary data on common variants from two large case-control GWAS of European ancestry on Type 2 Diabetes (T2D) and on Prostate Cancer (PrCa). Many previous studies have reported an inverse association of these two chronic diseases suggesting shared risk factors; however, shared genetic mechanisms underlying this T2D-PrCa association is poorly understood. We replicate some candidate and known shared genes, and identify a number of novel shared genetic regions.

## Material and methods

### Model and notation

Consider two genome-wide studies of traits *Y*_1_ and *Y*_2_ on *n*_1_ and *n*_2_ individuals respectively who were genotyped and/or imputed or sequenced at *p* genetic variants. Assume *n*_1_ individuals are independent of *n*_2_ individuals, with no overlapping samples between the studies. Let ***Y***_*k*_ and ***X***_*k*_ be the vectors of *k*-th trait values and genotypes at a given genetic variant respectively on all *n*_*k*_ individuals (*k* = 1, 2). For the ease of explanation, we will assume the two traits are binary (e.g., case-control traits); however, our approach, being based on summary statistics, is applicable to two qualitative and/or quantitative traits. An individual’s outcome or trait can take value 0 for controls or 1 for cases. If the genetic variant under consideration is a bi-allelic single nucleotide polymorphism (SNP), an individual’s genotype can take values 0, 1 or 2 depending on the number of copies of minor alleles at the SNP. If the variant is imputed, the genotypic value will range between 0 and 2. For simplicity, we assume there is no covariate. Note, this assumption can be easily relaxed by considering trait residuals (obtained from regressing the covariates on the trait) instead of the raw trait values. Although residualizing outcome data is not standard, previous studies have shown that it does not affect validity of genetic association tests [30–32].

The typical approach in a GWAS is to test for association of each genetic variant with the trait, and report the estimated genetic effect sizes, their standard errors and the corresponding p-values for all genetic variants (often referred to as ‘summary statistics’). For a given genetic variant, the marginal model for outcome data is

$$\begin{array}{c} \text{logit}(\text{P}(Y_{k}=1|X_{k}))=\alpha_{k}+\beta_{k}X_{k} \end{array}$$ (1)

where *β*_*k*_ is the genetic effect on the *k*-th trait (*k* = 1, 2). The null hypothesis of no association of the genetic variant with the *k*-th trait corresponds to $H_{0}^{\left(k\right)}:\;\beta_{k}=0$. The Wald test statistic $Z_{k}={\hat{\beta }}_{k}/{\hat{\sigma }}_{k}$ is used to test $H_{0}^{\left(k\right)}$, where ${\hat{\beta }}_{k}$ is the maximum likelihood estimate (MLE) of *β*_*k*_ and ${\hat{\sigma }}_{k}=\hat{\mathrm{se}}\left({\hat{\beta }}_{k}\right)$ is its estimated standard error. For common variants, the *Z*-score (*Z*_*k*_) has an asymptotic *N*(0, 1) distribution under the null $H_{0}^{\left(k\right)}$. Since the two studies are assumed to be independent, *Z*_1_ and *Z*_2_ are expected to be independently distributed. It is to be noted that the *Z*-scores can also be obtained under any other genetic model (e.g., dominant or recessive), and the following methodological development is still applicable.

### Statistical framework for a formal testing of pleiotropy

#### Defining the null hypothesis

The conventional cross-phenotype association methods test the global null hypothesis that none of the traits is associated with the given genetic variant (i.e., *β*_1_ = *β*_2_ = 0). Rejection of this global null can be due to one associated trait (*β*_1_ ≠ 0, *β*_2_ = 0 or *β*_1_ = 0, *β*_2_ ≠ 0) or both (*β*_1_ ≠ 0, *β*_2_ ≠ 0). Here, we are interested in identifying the genetic variants that are associated with both the traits or outcomes (i.e., pleiotropy). The effects of such a genetic variant on the traits may or may not be equal. Formally, our null hypothesis of no pleiotropy is *H*_0_: *at most 1 trait is associated with the genetic variant* while the alternative hypothesis is *H*_*a*_: *both traits are associated*.

#### A simple approach for testing pleiotropy

Mathematically, our null hypothesis of no pleiotropy is a composite null hypothesis *H*_0_: *H*_00_ ∪ *H*_01_ ∪ *H*_02_ while the alternative hypothesis is $H_{a}:\;H_{00}^{c}\cap H_{01}^{c}\cap H_{02}^{c}$, where *H*_00_: *β*_1_ = 0 = *β*_2_, *H*_01_: *β*_1_ = 0, *β*_2_ ≠ 0, *H*_02_: *β*_1_ ≠ 0, *β*_2_ = 0 and $A^{c}$ denotes the complement of set A. Thus, the alternative hypothesis is simply *H*_a_: *β*_1_ ≠ 0, *β*_2_ = 0 (the situation we are interested in identifying). This is a special two-parameter case of the intersection-union principle of statistical hypothesis testing. A level-*α* intersection-union test (IUT) [33] of *H*_0_ vs. *H*_*a*_ is, reject *H*_0_ if a level-*α* test rejects *H*_0*k*_ for every *k* = 1, 2. Consequently, the p-value for the IUT ≤ maximum of the p-values for testing $H_{0}^{\left(k\right)}:\;\beta_{k}=0$ vs. $H_{a}^{\left(k\right)}:\;\beta_{k}\neq 0$. Thus, an approximate conservative p-value of the IUT is max{*p*_1_, *p*_2_}, where *p*_*k*_ is the p-value corresponding to the test statistic *Z*_*k*_ (*k* = 1, 2) for model in Eq 1. We refer to this approximate test as ‘maxP’ in our figures and tables.

#### Other suitable approaches for testing pleiotropy

Observe that our null hypothesis of no pleiotropy can simply be written as *H*_0_: *β*_1_ *β*_2_ = 0 vs. the alternative hypothesis *H*_a_: *β*_1_ *β*_2_ ≠ 0. This immediately reminds us of the product of coefficients hypothesis tests for the significance of mediation effects in epidemiology [34]. It involves constructing test statistics by dividing ${\hat{\beta }}_{1}{\hat{\beta }}_{2}$ by its standard error, and comparing the observed value of the test statistic to a standard normal distribution. Several variants of the standard error of ${\hat{\beta }}_{1}{\hat{\beta }}_{2}$ are used based on different assumptions and order of derivatives in the approximations. If Sobel’s approach [34, 35] is used in our context to test *H*_0_, the test statistic is $Z_{1}Z_{2}/\sqrt{Z_{1}^{2}+Z_{2}^{2}}$, which uses an asymptotic *N*(0, 1) distribution as its null distribution.

In the context of genome-wide mediation analysis, the normal approximation of Sobel’s method depends on a condition that only holds if at least one of the mediation coefficients is non-zero [36]. In the context of our pleiotropy test in GWAS, we expect most genetic variants to be not associated with either of the traits (i.e., we expect the global null *H*_00_ to be true for most genetic variants). As a consequence of sparse signals and hence the breakdown of condition for asymptotic normality of Sobel’s method, testing pleiotropy using Sobel’s method fails to control type I error and lacks power to detect pleiotropic effects of a genetic variant. In the mediation literature, as an alternative to Sobel’s method, [36] proposed a modified p-value calculation for the test of estimated mediation effect that maintains appropriate type I error under the assumption that most of the significance tests of mediation are conducted under the global null that both coefficients are zero. In this article, we borrow Huang’s approach [36] from mediation analysis to propose a new single-variant test of pleiotropy of two traits in GWAS. Our approach for identifying pleiotropic variants is particularly useful for characterizing genetic overlap between two disease traits from case-control GWAS at a variant level.

### Our proposed test of pleiotropy: PLACO

#### Two independent traits

Suppose the global null *H*_00_ holds with probability *π*_00_ under which the single-trait test statistics *Z*_1_ and *Z*_2_ have asymptotic standard normal distributions. Further assume that the sub-null hypothesis *H*_01_ holds with probability *π*_01_ under which *Z*_1_ has a standard normal distribution and *Z*_2_ has a conditional *N*(*μ*_2_, 1) distribution given the mean parameter *μ*_2_. We assume a $N(0,\tau_{2}^{2})$ distribution for *μ*_2_. Similarly, assume that the sub-null hypothesis *H*_02_ holds with probability *π*_02_ and *Z*_2_ ∼ *N*(0, 1) while *Z*_1_|*μ*_1_ ∼ *N*(*μ*_1_, 1), where $\mu_{1}∼N\left(0,\tau_{1}^{2}\right)$.

In other words, we are assuming (a) *Z*_1_ and *Z*_2_ are independent *N*(0, 1) variables under *H*_00_; (b) *Z*_1_ and *Z*_2_ are independent *N*(0, 1) and $N(0,1+\tau_{2}^{2})$ variables respectively under *H*_01_; and (c) *Z*_1_ and *Z*_2_ are independent $N(0,1+\tau_{1}^{2})$ and *N*(0, 1) variables respectively under *H*_02_. Consequently, the products *Z*_1_ *Z*_2_, $Z_{1}\frac{Z_{2}}{\sqrt{1+\tau_{2}^{2}}}$ and $\frac{Z_{1}}{\sqrt{1+\tau_{1}^{2}}}Z_{2}$ have normal product distributions under *H*_00_, *H*_01_ and *H*_02_ respectively (assuming the parameters *τ*_1_ and *τ*_2_ are known). The (symmetric) normal product distribution is given by the probability density function (p.d.f.) $f\left(x\right)=K_{0}\left(|x|\right)/\pi$, − ∞ < *x* < ∞, where $K_{0}\left(.\right)$ is the modified Bessel function of the second kind with order 0 [37].

The p-value (two-tailed) for testing *H*_0_: *β*_1_ *β*_2_ = 0 (no pleiotropy) against *H*_a_: *β*_1_ *β*_2_ ≠ 0 using the product of *Z*-scores as our test statistic is given by

$$\begin{array}{cc} p_{Z_{1}Z_{2}} & =2\times {\text{P}}_{H_{0}}(Z_{1}Z_{2}>|z_{1}z_{2}\left|\right)=2\times \sum_{k=0}^{2}\text{P}\left(H_{0k}\right)\;{\text{P}}_{H_{0k}}(Z_{1}Z_{2}>|z_{1}z_{2}\left|\right)\\ & =\pi_{00}F\left(z_{1}z_{2}\right)+\pi_{01}F(z_{1}z_{2}/\sqrt{1+\tau_{2}^{2}})+\pi_{02}F(z_{1}z_{2}/\sqrt{1+\tau_{1}^{2}}) \end{array}$$ (2)

where *z*_1_ and *z*_2_ are the observed *Z*-scores for the two traits at a given genetic variant, and $F\left(u\right)=2\int_{\left|u\right|}^{\infty }f\left(x\right)dx$ is the two-sided tail probability of a normal product distribution at value *u*. Observe that the analytical form for PLACO p-value in Eq 2 contains unknown parameters *π*_00_, *π*_01_, *π*_02_, *τ*_1_ and *τ*_2_. One can estimate these parameters only once under the null using the GWAS summary statistics on the millions of genetic variants genome-wide and assume they are known. However, this p-value evaluation approach is sensitive to these parameter estimates and can be quite conservative at genome-wide levels (Section A of S1 Appendix). Instead we will use an approximate asymptotic p-value to test the null hypothesis of no pleiotropy.

#### Asymptotic approximation of PLACO p-value

The PLACO p-value in Eq 2 can be approximated as

$$\begin{array}{c} {\hat{p}}_{Z_{1}Z_{2}}=F(z_{1}z_{2}/\sqrt{\mathrm{Var}(Z_{1})})+F(z_{1}z_{2}/\sqrt{\mathrm{Var}(Z_{2})})-F(z_{1}z_{2}) \end{array}$$ (3)

where $\mathrm{Var}\left(Z_{1}\right)=1+\pi_{02}\tau_{1}^{2}$ and similarly Var(*Z*_2_) are the estimated marginal variances of the *Z*-scores under the hierarchical model we assumed [36]. This can be implemented using our R [38] program PLACO (https://github.com/RayDebashree/PLACO). Details of the estimation of parameters needed for calculating this approximate p-value are provided in Section A of S1 Appendix. The approximate p-value ${\hat{p}}_{Z_{1}Z_{2}}$ remains unchanged when mixture normal distributions or uniform distributions for the mean parameters *μ*_1_ and *μ*_2_ (under *H*_02_ and *H*_01_ respectively) are assumed [36].

#### Adjusting PLACO for correlation across GWAS

The above formulation of PLACO assumes that the *Z*-scores for the two traits are independent. While the independence of the effects ${\hat{\beta }}_{1}$ and ${\hat{\beta }}_{2}$, and consequently the *Z*-scores, is guaranteed in a mediation analysis assuming there is no unmeasured confounding [39], it is not guaranteed for a pleiotropy analysis. If the two traits come from studies with overlapping samples, either partially (e.g. studies with shared controls [40, 41]) or completely, then the *Z*-scores will be correlated [42] and may lead to inflated p-values or spurious signals if the correlation is not accounted for in the pleiotropic analysis.

For two outcomes from two case-control studies, the correlation between the *Z*-scores is $\rho \approx (n_{12,\text{control}}\sqrt{\frac{n_{1,\text{case}}n_{2,\text{case}}}{n_{1,\text{control}}n_{2,\text{control}}}}+n_{12,\text{case}}\sqrt{\frac{n_{1,\text{control}}n_{2,\text{control}}}{n_{1,\text{case}}n_{2,\text{case}}}})/\sqrt{n_{1}n_{2}}$ under the global null of no association, ignoring the variation due to $\hat{\mathrm{se}}\left({\hat{\beta }}_{k}\right)$’s, where *n*_*k*, case_ and *n*_*k*, control_ are respectively the number of cases and the number of controls in the study for *k*-th outcome, and *n*_12, control_ (*n*_12, case_) is the number of shared controls (cases) between the two studies [42]. In reality, the cases in two case-control studies are almost always independent and the control group in each study is frequently at least as large as the case group. The correlation *ρ*, thus, ranges between 0 and 0.5, where the maximum is reached when there are equal number of cases and controls in each study, both studies have the same sample size and all the controls are shared (Section B of S1 Appendix). For two continuous traits, the correlation between the *Z*-scores under the global null of no association is $\rho =\mathrm{corr}\left(Z_{1},Z_{2}\right)\approx \frac{n_{12}}{\sqrt{n_{1}n_{2}}}\mathrm{corr}\left(Y_{1},Y_{2}\right)$, where *n*_12_ is the total number of overlapping samples (i.e., individuals with measurements on both traits) and *n*_1_, *n*_2_ are the respective sample sizes of the two traits [42].

The number of overlapping samples between studies/traits may not be known when only GWAS summary data are available. In such a situation, one can estimate the correlation parameter *ρ* by the Pearson correlation of the *Z*-scores for the genetic variants with “no effect” on any trait. For a real dataset, the truth about which genetic variants have “no effect” is unknown. We choose the genetic variants that do not exceed a pre-defined significance threshold (say, genetic variants with single-trait p-value > 10^−4^) for any trait to estimate the correlation *ρ* between *Z*-scores [43]. One may also use cross-trait LD-score regression [44] to estimate *ρ*; however we did not find appreciable differences between GWAS results obtained using estimates from these two approaches [13]. Irrespective of the approach, this estimation is done only once, as implemented in PLACO software, before applying PLACO genome-wide. If **Z** = (*Z*_1_, *Z*_2_)′ be the vector of *Z*-scores for a given genetic variant and $\hat{R}=(\begin{array}{cc} 1 & \hat{\rho }\\ \hat{\rho } & 1 \end{array})$ be the estimated correlation matrix, one needs to de-correlate the *Z*-scores as ***Z***^decor^ = ***R***^−1/2^ ***Z*** so that $Z_{1}^{\mathrm{decor}}$ and $Z_{2}^{\mathrm{decor}}$ are uncorrelated. PLACO, as described before, can now be applied on these de-correlated *Z*-scores to test for pleiotropy of two correlated traits. However, we found from our simulation experiments that PLACO is an appropriate test of pleiotropy of two independent or moderately correlated traits, and may show inflated type I error for strongly correlated traits or when studies share more than half of their subjects.

### Simulation experiments

To evaluate operating characteristics of PLACO as a test for pleiotropy, we conduct simulation experiments in R [38]. We consider three broad simulation settings: one where we have traits from independent case-control studies, another with traits from case-control studies with shared controls, and the other with correlated traits from quantitative studies. For simplicity, we do not simulate any covariate or confounder. We simulate unrelated individuals and 10 million independent bi-allelic genetic variants in Hardy-Weinberg equilibrium with a fixed population-level minor allele frequency (MAF) 5%. We assume the commonly used additive genetic model in our simulations. Since we need multiple independent replicates to assess type I error control and power at stringent error thresholds, we generate the genetic variants independently. Subsequently, we calculate estimated type I error (power) by averaging over the number of independent null (non-null) variants identified as having significant pleiotropic effect on both traits at a fixed significance level *α*.

Out of the 10 million genetic variants, we assume 99% of variants to be under the global null of no association *H*_00_ (i.e., none of the two traits is associated with these genetic variants), 0.5% variants under the sub-null *H*_01_ (i.e., only the second trait is associated with these genetic variants), 0.4% variants under the sub-null *H*_02_ (i.e., only the first trait is associated with these genetic variants), and 0.1% variants under the alternative *H*_*a*_ (i.e., these genetic variants have pleiotropic effects on both traits). Thus, our simulated dataset has 9.99 million null variants to estimate type I error and 10, 000 non-null variants to estimate statistical power. Note, we have explored additional simulation settings such as those with higher proportion of variants associated with at least one trait or with larger MAF of variants; the details and results of which are provided in Section C of S1 Appendix.

#### Scenario I: Traits from two independent case-control studies

We simulate the two case-control studies such that the individuals in one study are independent of the other. We consider situations where the two studies have either comparable (1:1) or unbalanced (4:1) sample sizes. In other words, either the two studies have equal sample sizes (*n*_1_ = *n*_2_ = 2000) or the first study on the first trait is 4 times larger than the second study on the second trait (*n*_1_ = 8000, *n*_2_ = 2000). We assume a case-control ratio of 1:1 in each study, and a baseline disease prevalence of 15% and 10% for the first and the second disease trait respectively. Our generative model, described in Section C of S1 Appendix, has been widely used before [45–47] and is distinct from the hierarchical model assumed by PLACO. In this scenario, we compare type I error and power of Sobel’s approach, maxP, and PLACO to detect pleiotropy of the two independent case-control outcomes. Among the existing variant-level Bayesian pleiotropy methods applicable on a genome-wide scale, while both GPA and conditional FDR approaches are the most similar to PLACO in terms of the research question, we choose to compare PLACO with only GPA since GPA was previously shown to be superior to conditional FDR approach in most scenarios [23]. We keep this comparison separate from the main results because frequentist and Bayesian approaches are not directly comparable; moreover, PLACO aims to control FWER while GPA uses FDR control. The null genetic variants with non-zero effect on one trait only are assumed to have an odds ratio (OR) of 1.15 for the associated trait. For the non-null variants used to estimate power, we consider different choices of the two ORs to incorporate traits with genetic effects of varying directions and/or magnitudes.

#### Scenario II: Traits from two case-control studies with overlapping controls

We assume either 20%, 40%, 80% or 100% of the controls are shared, assuming equal number of controls in the two studies. Our generative model is the same as used in Scenario I. Here, we compare type I error of Sobel’s approach, maxP, and PLACO with and without correction for sample overlap. Evaluating power in this scenario is redundant since the power will depend on the total number of independent samples, which we explore in Scenario I. For implementing PLACO that accounts for the overlap, we assume the number of overlapping samples is not available to calculate correlation through the Lin-Sullivan approach [42], and instead estimate the Pearson correlation of the *Z*-scores.

#### Scenario III: Two correlated traits from a study of quantitative traits

We simulate a single study with measurements on two correlated quantitative traits measured either on the same individuals (*n*_1_ = *n*_2_ = 2000) or the first trait is measured on many additional individuals (*n*_1_ = 8000, *n*_2_ = 2000). We vary both the strength and the direction of pairwise trait correlation: *ρ*_*trait*_ = {−0.9, −0.4, 0, 0.4, 0.9}. The null genetic variants with non-zero effect on one trait only are assumed to explain 0.1% of the variance of the associated trait. The generative model is the same as before except that a bivariate normal model with means 0, variances 1, and pairwise correlation *ρ*_*trait*_ is used to simulate the quantitative traits. In this scenario too, we only compare type I error of Sobel’s approach, maxP, and PLACO (with and without correction for correlation), and do not evaluate power.

### Application to T2D and PrCa GWAS summary data

Many epidemiologic studies [48–52] of T2D and PrCa have reported association between these two diseases, suggesting shared risk factors. A few studies [53–56] have been undertaken to identify shared genetic risk factors underlying this T2D-PrCa association. To elucidate shared genetic mechanisms between these two diseases, which is still poorly understood, we use our statistical approach PLACO on summary data from two of the largest and most recent GWAS of T2D and of PrCa in individuals of European ancestry.

Xue et al. [57] meta-analyzed 62,892 T2D cases and 596,424 controls from three large GWAS datasets of European ancestry (DIAGRAM [58], GERA [59] and UK Biobank [60]). The authors reported summary statistics on 5,053,015 genotyped (from GWAS chip and Metabochip) and imputed autosomal SNPs (GRCh37/hg19) with MAF ≥1% that were common to the three datasets. All imputed SNPs have imputation info score ≥0.3. The reported summary statistics were obtained by fixed effects inverse-variance meta-analysis of GWAS summary statistics from each dataset after adjusting for study-specific covariates such as age, sex and principal components (PCs).

Schumacher et al. [61] meta-analyzed 79,194 PrCa cases and 61,112 controls from eight GWAS or high-density SNP panels of European ancestry imputed to 1000 Genomes Phase 3. All imputed SNPs have imputation *r*^2^ ≥ 0.3. The authors combined the per-allele odds ratios and standard errors, adjusted for PCs and study-relevant covariates, for the SNPs from the Illumina OncoArray and each GWAS by fixed effects inverse-variance meta-analysis. The summary statistics file contained information on 20,370,947 SNPs (GRCh37/hg19) across the autosomes and the X chromosome.

In this paper, we use the two sets of meta-analysis summary statistics of genetic association with T2D and with PrCa to detect shared common SNPs. Sources of these summary statistics are provided under Web resources. We remove any SNP with allele mismatch between the two datasets, and focus on the remaining 5, 041, 948 autosomal SNPs with MAF ≥1% that are available in both the studies. For a given SNP, we harmonize the same effect allele across the two studies so that *Z*-scores from the two datasets can be jointly analyzed appropriately using PLACO. From the effect estimates and the standard errors, we calculate the *Z*-scores, and remove SNPs with *Z*^2^>80 [62, 63] since extremely large effect sizes can disproportionately influence our analysis. The component studies underlying the T2D and the PrCa GWAS do not appear to overlap. The estimated correlation between the *Z*-scores from T2D and those from PrCa is approximately 0 as well.

To characterize the findings from PLACO, we clump all the significantly associated SNPs (*p*_PLACO_<5 × 10^-8^) in ±500 Kb radius and linkage disequilibrium (LD) threshold of *r*^2^>0.2 into a single genetic locus using FUMA [64] (SNP2GENE function, v1.3.5e). The gene annotations for all loci are based on proximity to the most significant/lead SNPs as mapped by FUMA. We perform different gene-set enrichment analyses using the GENE2FUNC function, where the genes were prioritized by FUMA based on the loci identified by PLACO. To provide additional evidence of sharing at these loci, we perform Bayesian colocalization test [65] of the PrCa and the T2D summary data using R package coloc (v3.2.1). This test computes 5 different overall posterior probabilities of the chosen region: ${\text{PP}}_{0}$ (posterior probability of no association with either disease), ${\text{PP}}_{1}$ (association with T2D, not with PrCa), ${\text{PP}}_{2}$ (association with PrCa, not with T2D), ${\text{PP}}_{3}$ (association with both T2D and PrCa due to two distinct causal SNPs) and ${\text{PP}}_{4}$ (association with both T2D and PrCa due to one common causal SNP). For each locus, we choose all the SNPs in ±200 Kb radius of the lead SNP and declare ‘convincing evidence’ of pleiotropic association of this locus if it shows ${\text{PP}}_{3}+{\text{PP}}_{4}\geq 0.9$ and ${\text{PP}}_{4}/{\text{PP}}_{3}\geq 3$ (cutoffs previously used elsewhere [66, 67]). For this analysis, we use the coloc.abf() function with default parameters and priors on the effect estimates and their variance estimates for the SNPs in the chosen region for each of T2D and PrCa. For the significant loci with convincing evidence of colocalization, we manually look up Open Targets Genetics platform [68] to gather information about diseases associated with nearby genes (selected options ‘genetic associations’, ‘pathways & systems biology’ and ‘RNA expression’), and on relevant mouse data if available. To characterize the regulatory effects of the significant pleiotropic signals, we perform whole blood *cis* expression quantitative trait locus (eQTL) analysis in FUMA using data from the eQTLGen Consortium [69], the largest publicly available meta-analysis of blood eQTLs based on >31,500 individuals. For *cis*-eQTL analysis, we additionally consider T2D-relevant tissues (liver, pancreas, adipose, skeletal muscle) [70] and PrCa-relevant tissue (prostate) from GTEx v8 [71].

## Results

### Simulation experiments: Type I error

#### Scenario I: Traits from two independent case-control studies

**Fig 1:**
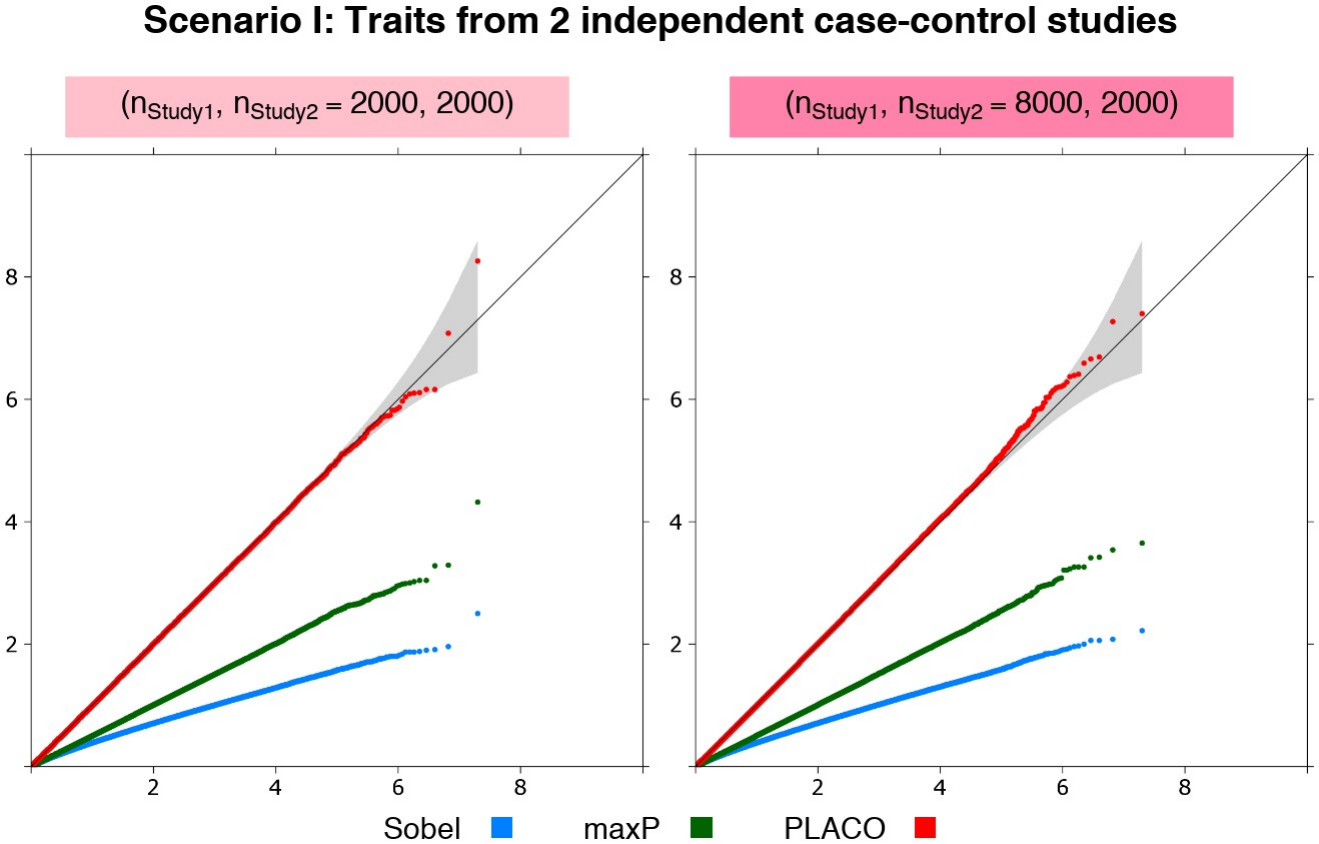
Scenario I: QQ plots for pleiotropic analysis of null data on traits from 2 independent case-control studies. Observed(−log_10_p-values) are plotted on the y-axis and Expected(−log_10_p-values) on the x-axis. Either each study has 1, 000 unrelated cases and 1, 000 unrelated controls, or Study 1 is 4 times that of Study 2, where Study 2 has 1, 000 unrelated cases and 1, 000 unrelated controls. Type I error performance of tests of pleiotropic effect of a genetic variant on the 2 traits is based on 9.99 million null variants with genetic effects that are either {*β*_1_ = 0 = *β*_2_} or {*β*_1_ = 0, *β*_2_ = log(1.15)} or {*β*_1_ = log(1.15), *β*_2_ = 0}. The gray shaded region represents a conservative 95% confidence interval for the expected distribution of p-values. P-values ≥10^-10^ are shown here.

Irrespective of whether the sample sizes of the two studies are same or widely different, PLACO has well-calibrated type I error at stringent significance levels (Fig 1). In comparison, the Sobel’s and maxP approaches are extremely conservative.

#### Scenario II: Traits from two case-control studies with overlapping controls

**Fig 2:**
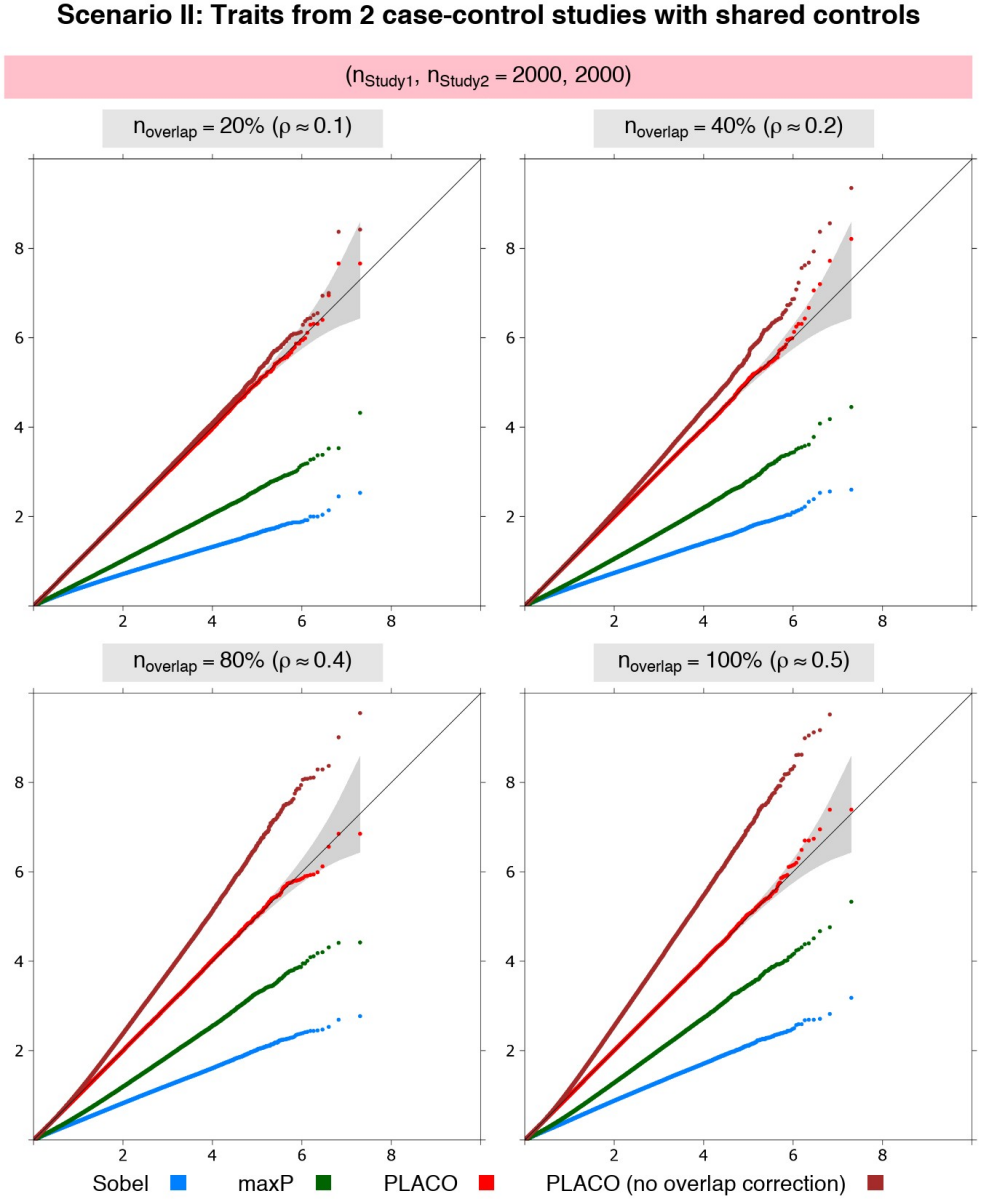
Scenario II: QQ plots for pleiotropic analysis of null data on traits from 2 case-control studies with different proportions of overlapping controls. Observed(−log_10_p-values) are plotted on the y-axis and Expected(−log_10_p-values) on the x-axis. Equal study sample size, and equal case-control size assumed in each study. Each study has 1, 000 unrelated cases and 1, 000 unrelated controls, of which either 20%, 40%, 80% or 100% of the controls are shared between the two studies. Type I error performance of tests of pleiotropic effect of a genetic variant on the 2 traits is based on 9.99 million null variants with genetic effects that are either {*β*_1_ = 0 = *β*_2_} or {*β*_1_ = 0, *β*_2_ = log(1.15)} or {*β*_1_ = log(1.15), *β*_2_ = 0}. The gray shaded region represents a conservative 95% confidence interval for the expected distribution of p-values. P-values ≥10^-10^ are shown here.

Regardless of the extent of control overlap in the two studies, PLACO exhibits appropriate type I error when correlation is accounted for in the analysis (Fig 2 and S1 Fig). We also note that if *Z*-scores are not decorrelated for studies with overlapping samples, pleiotropy analysis will likely show spurious association signals as indicated by the inflated ‘PLACO (no overlap correction)’ curve. The other approaches are still very conservative across all scenarios of overlap.

#### Scenario III: Two correlated traits from a study of quantitative traits

We find PLACO has well-calibrated type I error for moderately correlated traits irrespective of the direction of correlation between the traits, and has inflated type I error for strongly correlated traits (S2 Fig). Application of PLACO ignoring correlation will show spurious association signals. As before, the other approaches exhibit conservative behavior across all scenarios of pairwise trait correlation. The ‘maxP’ approach can, however, be less conservative for strongly correlated traits.

### Simulation experiments: Power

**Fig 3:**
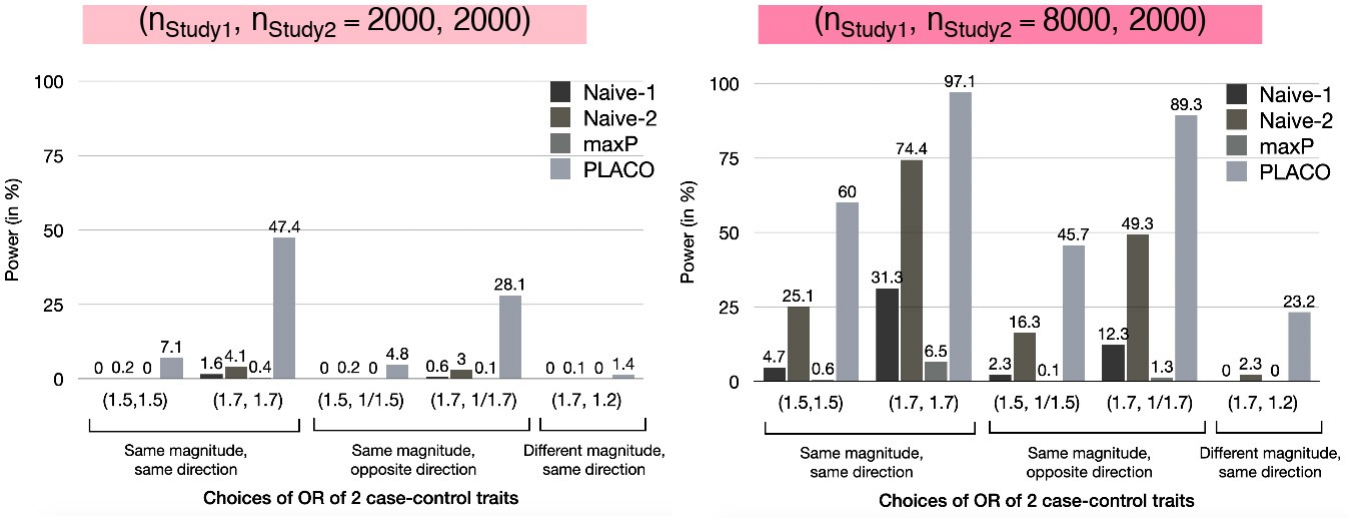
Scenario I: Power of PLACO, maxP and naive approaches at genome-wide significance level (5 × 10^−8^) for varying genetic effects of traits from 2 independent case-control studies. Sobel’s approch is excluded from this figure since it has <1% power across all scenarios. The first naive approach (‘Naive-1’) declares pleiotropic association when *p*_Trait1_<5 × 10^−8^ and *p*_Trait2_<5 × 10^−5^, while the second naive approach (‘Naive-2’) uses a more liberal criterion *p*_Trait1_<5 × 10^−8^ and *p*_Trait2_<5 × 10^−3^. Each study either has 1, 000 unrelated cases and 1, 000 unrelated controls, or Study 1 has 4 times sample size as Study 2, where Study 2 has 1, 000 unrelated cases and 1, 000 unrelated controls.

For benchmarking, we compare power of PLACO against Sobel’s and maxP, along with the naive approach of declaring pleiotropy when a variant reaches genome-wide significance for the first trait with the larger sample size and reaches a more liberal significance threshold for the second trait. We use two such naive approaches: one using criterion *p*_Trait1_<5 × 10^-8^, *p*_Trait2_<5 × 10^-5^ and the other *p*_Trait1_<5 × 10^-8^, *p*_Trait2_<5 × 10^-3^ (‘Naive-1’ and ‘Naive-2’ respectively in our figures). As reasoned before, comparing power under Scenario I is sufficient. Regardless of the magnitude and directions of pleiotropic association and the sample size differences between studies, PLACO has dramatically improved statistical power to detect pleiotropy compared to the naive approaches (Fig 3). The Sobel’s and maxP approaches especially lack power due to their very conservative type I error control.

### Simulation experiments: Comparison with an existing Bayesian approach

To make PLACO and GPA comparable to the extent possible, we use the Benjamini-Hochberg FDR [72] corrected PLACO p-values and 5% FDR threshold to declare significant pleiotropic association instead of using the FWER genome-wide threshold. For GPA, we use the association mapping results at global FDR threshold of 5% as provided by the R package GPA. It appears that PLACO is superior to GPA in terms of the number of discoveries made when fewer true pleiotropic variants are present genome-wide, especially if the pleiotropic effects are not very strong (S1 Table). This observation holds even for skewed sample sizes of the two traits (S2 Table).

### Application to T2D and PrCa GWAS summary data

#### Overview of joint T2D-PrCa locus level associations

**Fig 4:**
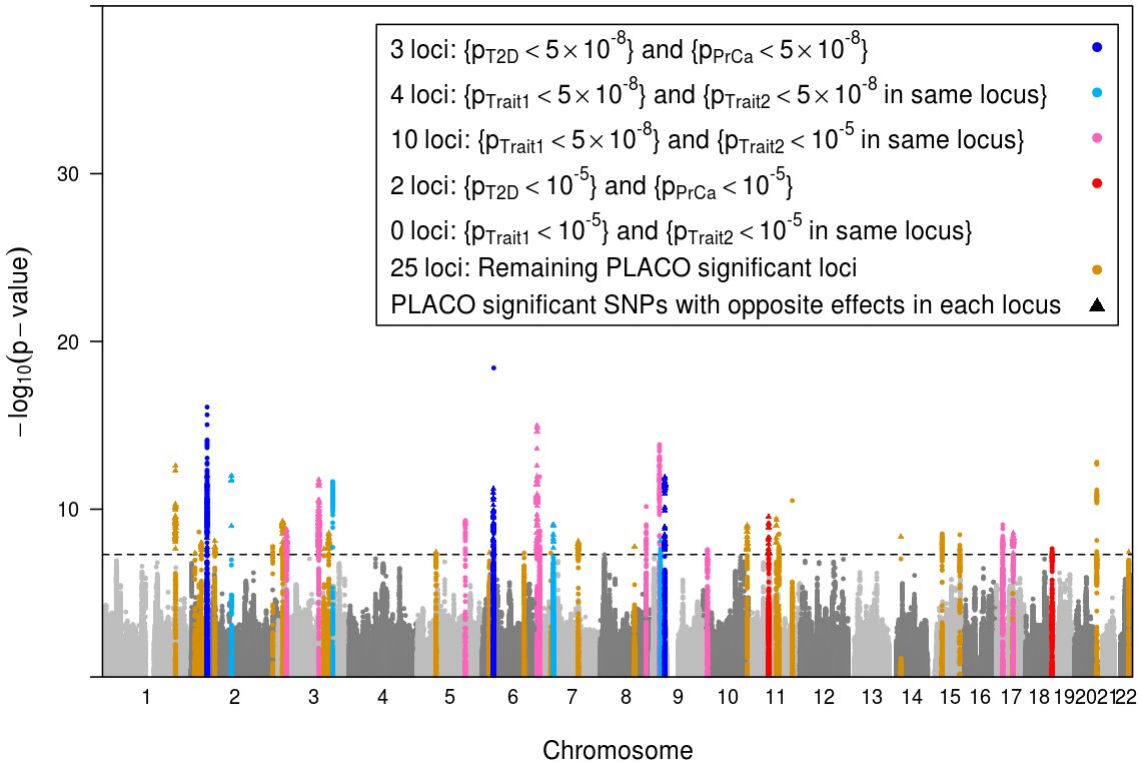
Manhattan plot of the PLACO p-values of pleiotropic association of common genetic variants with outcomes (traits) T2D and PrCa. The black horizontal dashed line corresponds to genome-wide significance level *α* = 5 × 10^−8^. The 44 loci with genome-wide significant pleiotropic lead SNP have been highlighted. A locus is defined by clumping SNPs in ±500 Kb radius around the lead SNP and with LD *r*^2^>0.2. Within each locus, if a PLACO significant SNP has genetic effects in opposite directions for T2D and PrCa, it is plotted as a solid triangle (24 such loci), else as a solid circle. Each identified pleiotropic locus is categorized (color-coded) as follows. Three loci harbor SNPs that are marginally genome-wide significant for both T2D and PrCa (single-trait *p*<5 × 10^−8^). Four loci contain SNPs that are marginally genome-wide significant for one disease, and in close proximity (i.e., in the same locus) with another SNP marginally genome-wide significant for the other disease. There are 10 loci where SNPs are marginally genome-wide significant for one disease and in close proximity with another SNP marginally suggestively significant (single-trait *p*<10^−5^) for the other disease. Two loci harbor SNPs that are marginally suggestively significant (but not genome-wide significant) for both T2D and PrCa. There is no locus that contains SNPs that are marginally suggestively significant (but not genome-wide significant) for one disease, and in close proximity with another SNP marginally suggestively significant (but not genome-wide significant) for the other disease. The rest of the 25 loci identified by PLACO contain SNPs that are not even marginally suggestively significant for either T2D or PrCa.

PLACO identified 1, 329 genome-wide significant SNPs that mapped to 44 distinct loci (Fig 4). The lead SNPs of 24 loci (55%) increase risk for one outcome while decreasing risk for the other. This observation is consistent with what observational studies [49, 73, 74] and genetic risk-score studies [54, 55] have reported before: an inverse association between T2D and PrCa. We define a locus as novel if there is no ‘previously associated SNP’ from GWAS catalog [75] (as of December 16, 2019) within ±500 Kb radius or in LD (*r*^2^>0.2) with our index SNP, the GWAS peak, from that locus. To define ‘previously associated SNP’ in our context of pleiotropy of T2D and PrCa, we looked for any SNP within each locus that is associated with both T2D-related trait (either of T2D, 2-hour glucose challenge, glucose level, glycated albumin, HbA1c, insulin level, pro-insulin level, insulin resistance, insulin response, or glycemic traits) and PrCa-related trait (either of PrCa or prostate-specific antigen levels). Since GWAS catalog includes exome-wide studies, we chose a slightly liberal exome-wide significance threshold of *p*<5 × 10^−7^ to define previously reported associations. We discovered 38 potentially novel loci, after liftover of GRCh38 genomic coordinates in GWAS catalog to hg19 using R package liftOver [76].

#### PLACO points to known and candidate shared genetic regions

GWAS catalog search reveals that 6 out of 44 loci near genes *THADA*, *BCL2L11*, *AC005355.2*, *PBX2* (in the major histo-compatibility complex or MHC region of 6p21), *JAZF1* and *CDKN2A/B* have been previously implicated in studies of both T2D and PrCa. In particular, *THADA* [51] (S3 Fig) and *JAZF1* [53] (S4 Fig) represent well-recognized shared genetic regions between T2D and PrCa. *HNF1B*, also known as *TCF2*, is another recognized shared gene [53, 77], which we fail to detect possibly because we excluded SNPs with extremely large effect sizes [62, 63] ($Z_{\text{PrCa}}^{2}>80$ for many SNPs positionally mapped in/near *HNF1B*), which may have weakened any signal in this region. Signals from PLACO point to candidate shared genes such as *PPARG* [55] (S5 Fig) and *CDKN2A* [51, 55] (S6 Fig). PLACO did not find enough evidence of shared genetic component in other previously explored genes such as *KCNQ1* [51] (S7 Fig) and *MTNR1B* [51] (S8 Fig).

#### Gene-set enrichment analysis

For further analysis, we exclude the 1 locus that lay in the MHC region of chromosome 6p21 because of strong SNP associations in this long-range and complex LD block that complicates fine-mapping efforts [70]. The 310 genes to which the 43 pleiotropic loci were mapped by FUMA are significantly enriched in GWAS catalog reported genes for PrCa, T2D and other T2D related traits (S9 Fig). When tested for tissue specificity against differentially expressed genes from GTEx v8 data across 53 tissue types, these genes are significantly enriched in pancreas (a T2D-relevant tissue) and whole-blood (S10 Fig). Analyses in other annotated gene sets from Molecular Signatures Database (MSigDB v7.0) [78] and in curated biological pathways from WikiPathways [79], and functional enrichment analyses are described in Section D of S1 Appendix.

#### Colocalization analysis

Bayesian colocalization tests of ±200 Kb region around the lead SNPs of the 43 loci reveal 26 lead SNPs as having the highest posterior probability of being associated with both PrCa and T2D (Table 1). Eight loci show convincing evidence of containing SNPs that are likely causal for both T2D and PrCa, 7 of which have the highest posterior probabilities of being causal SNPs and exhibit stronger signals of pleiotropic association compared to the single trait associations (Table 2). The lead SNP for the eighth locus, near *RGS17*, is 54 Kb away from the SNP with the highest causal probability (rs6932847), and both have similar PLACO p-value of pleiotropic association.

**Table 1:** The coloc colocalization posterior probability (${\text{PP}}_{4}$) for the lead SNPs from each of the 43 pleiotropic loci identified by PLACO.

Sl. no.	Lead SNP from PLACO analysis							coloc analysis of ±200kb around lead SNP						
								overall probabilities			SNP with highest causal probability			
	locus	position (hg19)	rsID	nearest gene	*p*_PLACO_	effect† direction	${\text{PP}}_{4}$	*n*_SNP_	${\text{PP}}_{3}+{\text{PP}}_{4}$	${\text{PP}}_{4}/{\text{PP}}_{3}$	rsID	position	*p*_PLACO_	${\text{PP}}_{4}$
1	1q32.1	204560677	rs6679717	*AL512306.3*	2.6 × 10^−13^	+ −	0.375	482	0.267	8		Same as lead SNP		
2	2p25.1	10094526	rs73913932	*GRHL1*	4.2 × 10^−8^	−+	0.394	909	0.33	40		Same as lead SNP		
3	2p24.1	20881840	rs2289081	*C2orf43*	2.3 × 10^−9^	+ +	1	690	0.192	14		Same as lead SNP		
4	2p23.3	27827092	rs12464616	*ZNF512*	9.9 × 10^−9^	−+	2 × 10^−6^	331	0.203	0.1	rs1260334	27748597	2.2 × 10^−6^	1
5	2p21	43797710	rs11904510	*THADA*	8.2 × 10^−17^	−−	0.168	809	1	0	rs10179648	43808065	9.0 × 10^−14^	0.434
6	2p14	65276452	rs1009358	*CEP68*	7.9 × 10^−9^	−+	0.75	792	0.407	15		Same as lead SNP		
7	2q13	111896243	rs17041869	*BCL2L11*	1.0 × 10^−12^	−+	0.446	626	0.994	1.2		Same as lead SNP		
8	2q36.3	227174983	rs2673148	*AC068138.1*	1.7 × 10^−8^	+ +	0.057	680	0.057	4.2	rs2673129	227139572	1.9 × 10^−8^	0.285
9	3p25.2	12276493	rs11709119	*PPARG*	5.3 × 10^−10^	−+	6 × 10^−4^	709	0.154	0.1	rs35000407	12351521	1.9 × 10^−4^	0.653
10	3p24.3	23284303	rs114460169	*UBE2E2*	1.7 × 10^−9^	−+	7 × 10^−6^	1179	0.672	0.0	rs1496653	23454790	8.7 × 10^−6^	1
11	3q13.2	113309149	rs6808932	*SIDT1*	1.8 × 10^−12^	+ −	0.394	728	0.879	0.4	rs12635148	113284208	2.6 × 10^−12^	0.605
12	3q21.3	128039895	rs11708733	*EEFSEC*	2.4 × 10^−8^	−+	9 × 10^−6^	488	0.023	0.6	rs2811478	127899624	7.2 × 10^−4^	0.071
13	3q23	141140366	rs6763927	*ZBTB38*	2.8 × 10^−9^	−+	0.174	504	0.923	5.3		Same as lead SNP		
14	3q25.1	152010142	rs76360965	*MBNL1*	2.3 × 10^−12^	−−	0.058	558	1	0.1		Same as lead SNP		
15	5q11.2	52058673	rs4530726	*ITGA1*	3.6 × 10^−8^	+ −	0.099	1026	0.826	7.1		Same as lead SNP		
16	5q31.1	133848917	rs10900829	*AC005355.2*	4.7 × 10^−10^	−−	0.109	358	0.877	1.9		Same as lead SNP		
17	6p22.3	20844151	rs9356756	*CDKAL1*	3.9 × 10^−8^	−+	0.064	849	0.043	0.3	rs9465883	20761335	1.3 × 10^−5^	0.189
18	6q22.1	117264990	rs1741652	*RFX6*	4.1 × 10^−8^	−−	10^−4^	716	0.1	0.1	rs682726	117104975	1.3 × 10^−3^	0.175
19	6q25.2	153394728	rs4385321	*RGS17*	1.1 × 10^−15^	+ −	0.17	1094	0.986	67	rs6932847	153448307	1.4 × 10^−15^	0.58
20	6q25.3	160683381	rs316025	*SLC22A2*	1.2 × 10^−12^	+ +	0.997	655	0.709	1.1		Same as lead SNP		
21	7p15.3	21012144	rs6944344	*LINC01162*	4.2 × 10^−8^	+ +	0.697	772	0.055	3.3		Same as lead SNP		
22	7p15.1	28028432	rs38514	*JAZF1*	8.3 × 10^−10^	+ −	0.366	626	1	0		Same as lead SNP		
23	7q21.3	97754074	rs73404162	*LMTK2*	8.4 × 10^−9^	−+	7 × 10^−8^	577	0.215	0.1	rs12667763	97668012	7.0 × 10^−8^	0.704
24	8q22.1	95739642	rs67763258	*DPY19L4*	1.7 × 10^−8^	−+	0.507	1019	0.368	7.8		Same as lead SNP		
25	8q24.21	128391412	rs62516032	*CASC8*	6.9 × 10^−11^	−−	0.093	550	0.518	0.0	rs1962471	128281708	1.6 × 10^−6^	0.197
26	9p22.1	19064129	rs13287517	*HAUS6*	1.4 × 10^−14^	+ +	0.379	1322	0.999	30		Same as lead SNP		
27	9p21.3	22003223	rs3217992	*CDKN2A/B*	7.5 × 10^−9^	+ −	10^−4^	482	1	0	rs1063192	22003367	1.7 × 10^−6^	0.739
28	9p13.3	34025640	rs1758632	*UBAP2*	1.2 × 10^−12^	−+	0.065	511	1	15		Same as lead SNP		
29	10p13	12208307	rs1053403	*NUDT5*	2.6 × 10^−8^	+ +	10^−8^	646	0.744	0.0	rs11257655	12307894	3.8 × 10^−7^	0.869
30	10q26.12	123038897	rs12413648	*LINC01153*	9.2 × 10^−10^	+ −	0.15	714	0.651	3.4		Same as lead SNP		
31	11p11.2	47461693	rs7103835	*RAPSN*	2.8 × 10^−10^	−+	0.503	467	0.992	11		Same as lead SNP		
32	11q13.3	68894753	rs12284087	*RP11-554A11.7*	3.9 × 10^−10^	+ +	0.67	547	0.179	1.4		Same as lead SNP		
33	11q13.5	76257215	rs3753051	*C11orf30*	3.0 × 10^−9^	−−	0.123	714	0.262	2	rs17749618	76251818	3.2 × 10^−9^	0.129
34	11q23.2	113807181	rs11214775	*HTR3A/B*	3.1 × 10^−11^	−−	1	640	0.723	97		Same as lead SNP		
35	14q13.1	33302882	rs17522122	*AKAP6*	4.4 × 10^−9^	+ −	0.973	787	0.94	980		Same as lead SNP		
36	15q15.1	40881116	rs10400825	*KNL1*	3.0 × 10^−9^	−−	0.058	625	0.908	11		Same as lead SNP		
37	15q26.1	90429148	rs12912009	*AP3S2*	3.3 × 10^−9^	+ +	0.222	520	0.382	2.8		Same as lead SNP		
38	17p11.2	17724789	rs11656665	*SREBF1*	8.4 × 10^−10^	+ +	0.289	412	0.951	0.4		Same as lead SNP		
39	17q21.32	45885756	rs9911983	*OSBPL7*	4.8 × 10^−8^	−+	0.939	683	0.707	13		Same as lead SNP		
40	17q21.32	47037024	rs11079847	*GIP*	2.7 × 10^−9^	−+	0.016	667	0.843	0.1	rs9894220	46989154	7.4 × 10^−9^	0.172
41	18q23	74562251	rs7236466	*ZNF236*	2.3 × 10^−8^	+ +	0.1	880	0.949	14		Same as lead SNP		
42	20q13.33	62337406	rs6011040	*ARFRP1*	1.6 × 10^−13^	−−	0.367	281	0.724	22		Same as lead SNP		
43	22q13.1	40479811	rs9607685	*TNRC6B*	3.7 × 10^−8^	−+	0.035	393	0.114	5.7	rs34419824	40499103	1.6 × 10^−7^	0.267

^†^ The effect direction duplet reports the effect direction of T2D first, and then of PrCa for the chosen effect allele at the lead SNP.

A high ${\text{PP}}_{4}$ for a SNP indicates high probability of being the common causal SNP for both T2D and PrCa. SNPs with highest ${\text{PP}}_{4}$ within ±200 Kb of the lead SNPs are also reported.

**Table 2:** The potentially novel loci detected by PLACO and with convincing evidence (${\text{PP}}_{3}+{\text{PP}}_{4}\geq 0.9$ and ${\text{PP}}_{4}/{\text{PP}}_{3}\geq 3$) of being causal for both T2D and PrCa from colocalization analysis.

Locus no.	Lead SNP from PLACO analysis								Summary statistics for lead SNP				*p*_PLACO_
	locus	position hg19)	rsID	nearest gene	effect allele	other allele	effect allele freq.	CADD score	${\hat{\beta }}_{\text{T2D}}$	*p*_T2D_	${\hat{\beta }}_{\text{PrCa}}$	*p*_PrCa_	
13	3q23	141140366	rs6763927	*ZBTB38*	T	A	0.44	3.18	-0.0316	6.8 × 10^−5^	0.0459	8.5 × 10^−9^	2.8 × 10^−9^
19	6q25.2	153394728	rs4385321	*RGS17*	A	G	0.35	4.05	0.0352	2.8 × 10^−6^	-0.0724	2.7 × 10^−18^	1.1 × 10^−15^
26	9p22.1	19064129	rs13287517	*HAUS6*	C	G	0.39	0.44	0.0402	5.3 × 10^−7^	0.0609	7.1 × 10^−14^	1.4 × 10^−14^
28	9p13.3	34025640	rs1758632	*UBAP2*	C	G	0.38	1.24	-0.0491	1.4 × 10^−9^	0.0432	1.1 × 10^−7^	1.2 × 10^−12^
31	11p11.2	47461693	rs7103835	*RAPSN*	A	G	0.31	7.53	-0.0384	1.2 × 10^−6^	0.046	1.4 × 10^−7^	2.9 × 10^−10^
35	14q13.1	33302882	rs17522122	*AKAP6*	T	G	0.48	2.19	0.0403	5.2 × 10^−8^	-0.0337	4.0 × 10^−5^	4.4 × 10^−9^
36	15q15.1	40881116	rs10400825	*KNL1*	G	A	0.15	2.66	-0.0452	4.0 × 10^−5^	-0.0612	2.4 × 10^−8^	3.0 × 10^−9^
41	18q23	74562251	rs7236466	*ZNF236*	G	T	0.38	4.03	0.0368	3.8 × 10^−6^	0.0364	8.2 × 10^−6^	2.3 × 10^−8^

${\text{PP}}_{3}$ is the probability that the association of a SNP with both T2D and PrCa is due to two distinct causal SNPs; ${\text{PP}}_{4}$ is the probability that the association of a SNP with both T2D and PrCa is due to one common causal SNP.

#### Characterizing the 8 most interesting potentially novel pleiotropic loci

**Fig 5:**
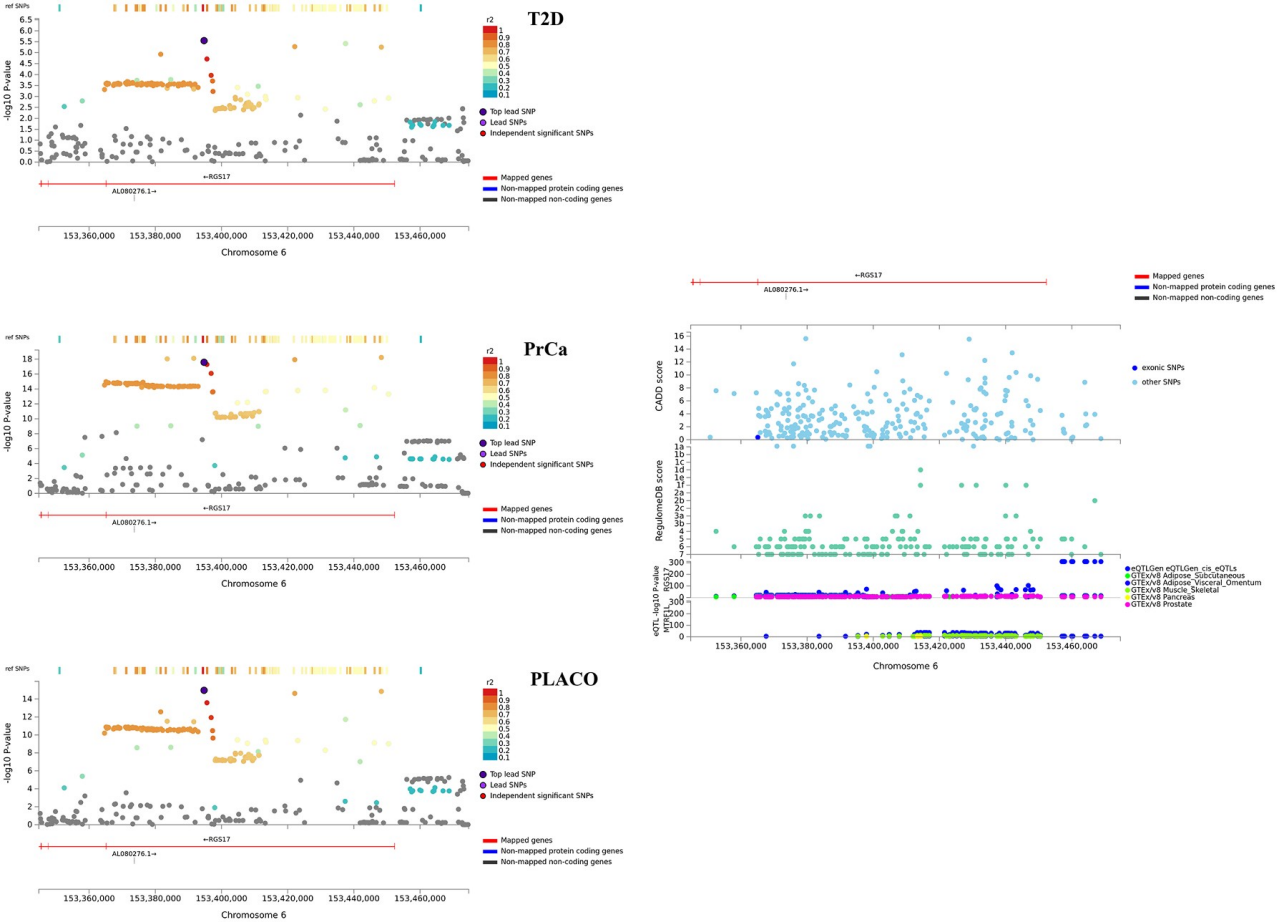
Regional association plot of significant pleiotropic locus near *RGS17* with annotations such as CADD scores, RegulomeDB scores, and *cis* eQTL association p-values from 6 tissues. Tissues considered are whole blood from eQTLGen Consortium; and adipose, liver, muscle-skeletal, pancreas, and prostate tissues from GTEx v8.

**Fig 6:**
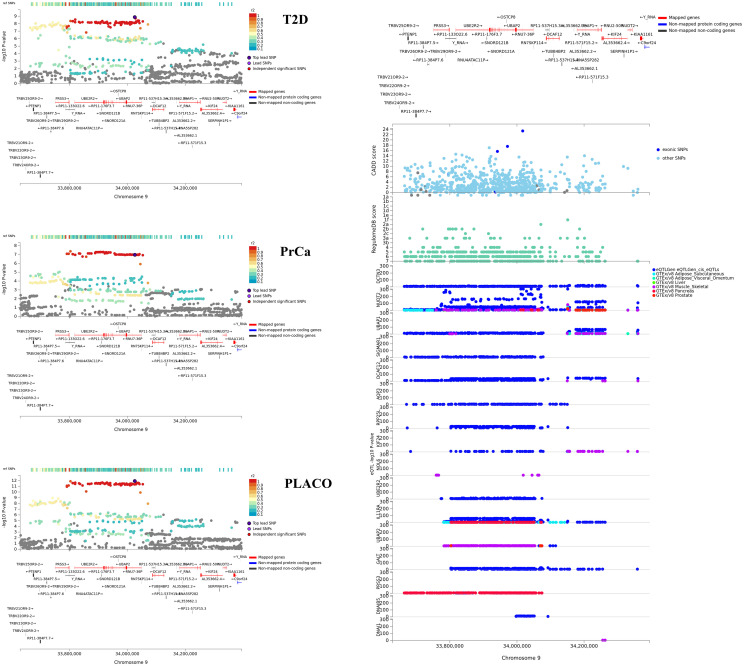
Regional association plot of significant pleiotropic locus near *UBAP2* with annotations such as CADD scores, RegulomeDB scores, and *cis* eQTL association p-values from 6 tissues. Tissues considered are whole blood from eQTLGen Consortium; and adipose, liver, muscle-skeletal, pancreas, and prostate tissues from GTEx v8.

The lead SNPs of 6 of the 8 potentially novel pleiotropic loci with convincing evidence from the colocalization analyses have effect alleles that increase risk for one disease while protecting from the other (Table 2). While the 8 loci contain *cis*-eQTLs in multiple T2D-relevant tissues (S11–S16 Figs), SNPs in the loci near *RGS17* (Fig 5) and *UBAP2* (Fig 6) show significant *cis*-eQTL associations in both T2D-relevant and PrCa-relevant tissues. In Open Targets Genetics, genes near the *ZBTB38*, *UBAP2* and *ZNF236* loci show associations with various cancers, diabetes and obesity (no relevant mouse data available for these genes). The *RGS17* locus show associations with various cancers, including PrCa and prostate neoplasm, and body mass index (BMI) but has no known associations with any T2D-related trait (no relevant mouse data available). Of particular interest are the *HAUS6* and the *RAPSN* loci. While *HAUS6* and its nearby genes *RRAGA* and *PLIN2* have various cancers (including PrCa) as associated diseases in Open Targets Genetics, one or more of them are related to metabolism phenotype, abnormal gluconeogenesis and hypoglycemia in mice. GWAS catalog search of these genes did not yield any known association result with any T2D-related trait. Similarly, the nearby gene *MADD* for the *RAPSN* locus has various cancers, neoplasms and glucose-related phenotypes as associated diseases in Open Targets Genetics; and is a recognized T2D gene, which when knocked out in mice, show impaired glucose tolerance, hyperglycemia and abnormal pancreatic beta cell morphology.

## Discussion

In this paper, we propose a formal statistical hypothesis test and a novel method, PLACO, to determine common pleiotropic or shared variants of two independent traits and show how it may well be applied to correlated traits or traits from studies with sample overlap. In our simulations involving qualitative and quantitative traits with unequal prevalences, unequal genetic effect sizes, unequal sample sizes—ranging from modest to large—and with/without overlapping samples, PLACO exhibits well-calibrated type I error. We find PLACO is powerful in detecting subtle genetic effects of pleiotropic variants that may or may not be in the same direction and that may be missed when each disease trait is analyzed separately (see some additional simulations in Section C of S1 Appendix). Statistical power is significantly improved when PLACO is used, compared to the naive approach that identifies pleiotropy when a genetic variant reaches genome-wide significance for the trait with larger sample size and reaches a more liberal threshold for the other. We also observe improved power over other existing approaches, both Bayesian and frequentist, in most scenarios. Based on our simulations, we advocate using PLACO on independent traits, or moderately correlated traits after decorrelating the *Z*-scores as described before.

We use the most recent publicly available case-control GWAS summary data on T2D and on PrCa in individuals of European ancestry to determine variants that influence risk to both these diseases. We identify several known and candidate shared genes, and detect a number of novel shared genetic regions near *ZBTB38* (3q23), *RGS17* (6q25.3), *HAUS6* (9p22.1), *UBAP2* (9p13.3), *RAPSN* (11p11.2), *AKAP6* (14q12), *KNL1* (15q15) and *ZNF236* (18q23). A recent study [80] showed a weak positive genetic correlation between T2D and PrCa. It is worth noting that the concept of genetic correlation is different from pleiotropy. For genetic correlation to be non-zero, the directions of effect of non-null variants must be consistently aligned [44]. Effect alleles of at least half of the significant SNPs identified by PLACO have opposite genetic effects on the two diseases, which supports many previous studies reporting inverse relationship between T2D and PrCa, and likely explains the weak genetic correlation in the previous study.

The key advantage of PLACO among existing frequentist approaches is not requiring individual-level data which makes it easily applicable to datasets for which only GWAS summary data are available. It does not require compute intensive permutations or Monte Carlo simulations to calculate p-value of simultaneous association of two traits with one genetic variant. We are conveniently using the asymptotic normality of MLE of genetic effects to get at the null distribution of the PLACO test statistic. The existence of an analytical form for PLACO p-value (Eq 2) and its approximation (Eq 3) makes it suitable for application on a genome-wide scale. While we have applied PLACO to summary statistics from population-based case-control GWAS, it may also be applied to two traits from family-based designs (e.g., disease traits from case-parent trio studies). For instance, family-based GWAS data from several study cohorts will soon be available under the cohort collaboration study, Environmental influences on Child Health Outcomes (ECHO, https://www.nih.gov/research-training/environmental-influences-child-health-outcomes-echo-program), to understand genetic underpinnings of pediatric outcomes. One important scientific question will be to identify genetic overlap of such outcomes (e.g., neurodevelopmental disorders, respiratory disorders), which PLACO can conveniently address, that too without having to pool individual-level data.

Our study and our statistical approach are not without limitations. PLACO requires genome-wide summary data to infer pleiotropic association of each variant, and cannot be used when summary data on only a handful of candidate genetic variants are available. Calculation of PLACO p-value requires parameter estimation using variants across the genome, and hence cannot be used to test pleiotropy of a set of variants known to be significantly associated with one trait. PLACO shows inflated type I error when the traits are strongly correlated even after using our decorrelation approach. The approximate PLACO p-value (${\hat{p}}_{Z_{1}Z_{2}}$) is a good approximation when the non-zero effect under *H*_01_ or *H*_02_ is small [36], else it may be inflated. Simply stated, if the effect of a genetic variant is very strong on one trait and has no effect on the other trait, the p-value reported by PLACO may be inflated and indicate a genome-wide significant result. We suggest that SNPs with marginal *Z*^2^>80 be removed before analysis, similar to suggestion for LD-score regression approaches. PLACO is a single-variant association test that is not expected to control type I error for genetic variants with low minor allele counts since the asymptotic normality of MLE assumption may be violated [13]. It is assumed that the summary statistics on which PLACO is applied are obtained after appropriately accounting for all confounding effects, including relatedness and population stratification. PLACO can only detect statistical association of a variant with two traits, referred to as ‘statistical pleiotropy’ [67], and cannot distinguish between the various types of pleiotropy: biological, mediated, spurious due to design artefacts or spurious due to strong LD between causal variants in different genes [1]. Notwithstanding these caveats, PLACO provides massive power gain over commonly used approaches, and shows promise in providing additional evidence for a shared genetic component between two traits.

## Supporting information

S1 Appendix Additional text and supporting information. It includes additional details on PLACO p-value calculation, simulation experiments, and analysis of T2D and PrCa datasets.
(PDF)

S1 Fig Scenario II: QQ plots for the pleiotropic analysis of null data on traits from 2 case-control studies with different proportions of overlapping controls. Observed(−log_10_p-values) are plotted on the y-axis and Expected(−log_10_p-values) on the x-axis. Unequal study sample size, and equal case-control size assumed in each study. Study 1 has 4, 000 unrelated cases and 4, 000 unrelated controls. Study 2 has 1, 000 unrelated cases and 1, 000 unrelated controls, of which either 20%, 40%, 80% or 100% of the controls are shared between the two studies. Type I error performance of tests of pleiotropic effect of a genetic variant on the 2 traits is based on 9.99 million null variants with genetic effects that are either {*β*_1_ = 0 = *β*_2_} or {*β*_1_ = 0, *β*_2_ = log(1.15)} or {*β*_1_ = log(1.15), *β*_2_ = 0}. The gray shaded region represents a conservative 95% confidence interval for the expected distribution of p-values.
(PDF)

S2 Fig Scenario III: QQ plots for the pleiotropic analysis of null data on 2 correlated traits where each trait is measured on the same 2, 000 individuals. Observed(−log_10_p-values) are plotted on the y-axis and Expected(−log_10_p-values) on the x-axis. Type I error performance of tests of pleiotropic effect of a genetic variant on the 2 traits is based on 9.99 million null variants with genetic effects that are either {*β*_1_ = 0 = *β*_2_} or {*β*_1_ = 0, *β*_2_ explains 0.1% of Trait 2 variance} or {*β*_1_ explains 0.1% of Trait 1 variance, *β*_2_ = 0}. The gray shaded region represents a conservative 95% confidence interval for the expected distribution of p-values. P-values ≥10^−12^ are shown here.
(PDF)

S3 Fig Locuszoom plots of association p-values for variants in and around gene *THADA*. (PDF)

S4 Fig Locuszoom plots of association p-values for variants in and around gene *JAZF1*. (PDF)

S5 Fig Locuszoom plots of association p-values for variants in and around gene *PPARG*. (PDF)

S6 Fig Locuszoom plots of association p-values for variants in and around gene *CDKN2A*. (PDF)

S7 Fig Locuszoom plots of association p-values for variants in and around gene *KCNQ1*. (PDF)

S8 Fig Locuszoom plots of association p-values for variants in and around gene *MTNR1B*. (PDF)

S9 Fig Mapped genes (as done by FUMA) for the 43 pleiotropic loci detected by PLACO were tested for enrichment in GWAS catalog reported genes across diseases and traits. (PDF)

S10 Fig Mapped genes (as done by FUMA) for the 43 pleiotropic loci detected by PLACO were tested against each of the Differentially Expressed Gene (DEG) sets pre-calculated from GTEx v8 tissue data from 53 tissue types. (PDF)

S11 Fig Regional association plot of significant pleiotropic locus near *ZBTB38* with annotations such as CADD scores, RegulomeDB scores, and *cis* eQTL association p-values from 6 tissues. Tissues considered are whole blood from eQTLGen Consortium; and adipose, liver, muscle-skeletal, pancreas, and prostate tissues from GTEx v8.
(PDF)

S12 Fig Regional association plot of significant pleiotropic locus near *HAUS6* with annotations such as CADD scores, RegulomeDB scores, and *cis* eQTL association p-values from 6 tissues. Tissues considered are whole blood from eQTLGen Consortium; and adipose, liver, muscle-skeletal, pancreas, and prostate tissues from GTEx v8.
(PDF)

S13 Fig Regional association plot of significant pleiotropic locus near *RAPSN* with annotations such as CADD scores, RegulomeDB scores, and *cis* eQTL association p-values from 6 tissues. Tissues considered are whole blood from eQTLGen Consortium; and adipose, liver, muscle-skeletal, pancreas, and prostate tissues from GTEx v8.
(PDF)

S14 Fig Regional association plot of significant pleiotropic locus near *AKAP6* with annotations such as CADD scores, RegulomeDB scores, and *cis* eQTL association p-values from 6 tissues. Tissues considered are whole blood from eQTLGen Consortium; and adipose, liver, muscle-skeletal, pancreas, and prostate tissues from GTEx v8.
(PDF)

S15 Fig Regional association plot of significant pleiotropic locus near *KNL1* with annotations such as CADD scores, RegulomeDB scores, and *cis* eQTL association p-values from 6 tissues. Tissues considered are whole blood from eQTLGen Consortium; and adipose, liver, muscle-skeletal, pancreas, and prostate tissues from GTEx v8.
(PDF)

S16 Fig Regional association plot of significant pleiotropic locus near *ZNF236* with annotations such as CADD scores, RegulomeDB scores, and *cis* eQTL association p-values from 6 tissues. Tissues considered are whole blood from eQTLGen Consortium; and adipose, liver, muscle-skeletal, pancreas, and prostate tissues from GTEx v8.
(PDF)

S1 Table Scenario I: Comparison of PLACO and GPA in terms of error control and power for 2 independent case-control studies, where each study has 1,000 unrelated cases and 1,000 unrelated controls. Each study has 9.9 × 10^6^ null variants (i.e., variants under *H*_00_ or *H*_01_ or *H*_02_) and *m* non-null (pleiotropic) variants, where *m* takes values 0, 100, 300, 500, 1000, 3000, 5000 or 10000. Five different choices of odds ratios of association of *m* non-null variants with Traits 1 and 2 are considered. The total number of true positives (non-null variants) detected (#TP) and the total number of false positives detected (#FP) are reported. PLACO’s performance for both genome-wide threshold 5 × 10^−8^ (or equivalently family-wise error rate (FWER) of 5%) and global false discovery rate (FDR) of 5% are reported, while GPA’s performance is based on global FDR of 5%.
(PDF)

S2 Table Scenario I: Comparison of PLACO and GPA in terms of error control and power for 2 independent case-control studies, where Study 1 has 4 times sample size as Study 2, and Study 2 has 1, 000 unrelated cases and 1, 000 unrelated controls. Each study has 9.9 × 10^6^ null variants (i.e., variants under *H*_00_ or *H*_01_ or *H*_02_) and *m* non-null (pleiotropic) variants, where *m* takes values 0, 100, 300, 500, 1000, 3000, 5000 or 10000. Five different choices of odds ratios of association of *m* non-null variants with Traits 1 and 2 are considered. The total number of true positives (non-null variants) detected (#TP) and the total number of false positives detected (#FP) are reported. PLACO’s performance for both genome-wide threshold 5 × 10^−8^ (or equivalently family-wise error rate (FWER) of 5%) and global false discovery rate (FDR) of 5% are reported, while GPA’s performance is based on global FDR of 5%.
(PDF)

## References

[1] Solovieff N, Cotsapas C, Lee PH, Purcell SM, Smoller JW. Pleiotropy in complex traits: challenges and strategies. Nat Rev Genet. 2013;14(7):483–495. 10.1038/nrg3461 23752797 PMC4104202

[2] Sivakumaran S, Agakov F, Theodoratou E, Prendergast JG, Zgaga L, Manolio T, et al Abundant pleiotropy in human complex diseases and traits. Am J Hum Genet. 2011;89(5):607–618. 10.1016/j.ajhg.2011.10.004 22077970 PMC3213397

[3] Wu YH, Graff RE, Passarelli MN, Hoffman JD, Ziv E, Hoffmann TJ, et al Identification of pleiotropic cancer susceptibility variants from genome-wide association studies reveals functional characteristics. Cancer Epidemiol Biomarkers Prev. 2018;27(1):75–85. 10.1158/1055-9965.EPI-17-0516 29150481 PMC5760292

[4] Cotsapas C, Voight BF, Rossin E, Lage K, Neale BM, Wallace C, et al Pervasive sharing of genetic effects in autoimmune disease. PLoS Genet. 2011;7(8):e1002254 10.1371/journal.pgen.1002254 21852963 PMC3154137

[5] Cross-Disorder Group of the Psychiatric Genomics Consortium. Identification of risk loci with shared effects on five major psychiatric disorders: a genome-wide analysis. Lancet. 2013;381(9875):1371–1379. 10.1016/S0140-6736(12)62129-1 23453885 PMC3714010

[6] Amare AT, Vaez A, Hsu YH, Direk N, Kamali Z, Howard DM, et al Bivariate genome-wide association analyses of the broad depression phenotype combined with major depressive disorder, bipolar disorder or schizophrenia reveal eight novel genetic loci for depression. Mol Psychiatry. 2019; p. 1 10.1038/s41380-018-0336-6 30626913 PMC7303007

[7] Li R, Brockschmidt FF, Kiefer AK, Stefansson H, Nyholt DR, Song K, et al Six novel susceptibility Loci for early-onset androgenetic alopecia and their unexpected association with common diseases. PLoS Genet. 2012;8(5):e1002746 10.1371/journal.pgen.1002746 22693459 PMC3364959

[8] Hui KY, Fernandez-Hernandez H, Hu J, Schaffner A, Pankratz N, Hsu NY, et al Functional variants in the *LRRK2* gene confer shared effects on risk for Crohn’s disease and Parkinson’s disease. Sci Transl Med. 2018;10(423):eaai7795 10.1126/scitranslmed.aai7795 29321258 PMC6028002

[9] Pickrell JK, Berisa T, Liu JZ, Ségurel L, Tung JY, Hinds DA. Detection and interpretation of shared genetic influences on 42 human traits. Nat Genet. 2016;48(7):709–717. 10.1038/ng.3570 27182965 PMC5207801

[10] Yang C, Li C, Wang Q, Chung D, Zhao H. Implications of pleiotropy: challenges and opportunities for mining Big Data in biomedicine. Front Genet. 2015;6:229 10.3389/fgene.2015.00229 26175753 PMC4485215

[11] Gratten J, Visscher PM. Genetic pleiotropy in complex traits and diseases: implications for genomic medicine. Genome Med. 2016;8(1):78 10.1186/s13073-016-0332-x 27435222 PMC4952057

[12] Hackinger S, Zeggini E. Statistical methods to detect pleiotropy in human complex traits. Open Biol. 2017;7(11):170125 10.1098/rsob.170125 29093210 PMC5717338

[13] Ray D, Chatterjee N. Effect of non-normality and low count variants on cross-phenotype association tests in GWAS. Eur J Hum Genet. 2020;28:300–312. 10.1038/s41431-019-0514-2 31582815 PMC7028917

[14] Baker AR, Goodloe RJ, Larkin EK, Baechle DJ, Song YE, Phillips LS, et al. Multivariate association analysis of the components of metabolic syndrome from the Framingham Heart Study. In: BMC Proc. vol. 3. BioMed Central; 2009. p. S42. 10.1186/1753-6561-3-s7-s42 PMC2795941 20018034

[15] Inouye M, Ripatti S, Kettunen J, Lyytikäinen LP, Oksala N, Laurila PP, et al Novel Loci for metabolic networks and multi-tissue expression studies reveal genes for atherosclerosis. PLoS Genet. 2012;8(8):e1002907 10.1371/journal.pgen.1002907 22916037 PMC3420921

[16] Medina-Gomez C, Kemp JP, Dimou NL, Kreiner E, Chesi A, Zemel BS, et al Bivariate genome-wide association meta-analysis of pediatric musculoskeletal traits reveals pleiotropic effects at the *SREBF1/TOM1L2* locus. Nat Commun. 2017;8(1):121 10.1038/s41467-017-00108-3 28743860 PMC5527106

[17] Heid IM, Winkler TW. A multitrait GWAS sheds light on insulin resistance. Nat Genet. 2017;49(1):7 10.1038/ng.3758 28029160

[18] Shen X, Klarić L, Sharapov S, Mangino M, Ning Z, Wu D, et al Multivariate discovery and replication of five novel loci associated with immunoglobulin GN-glycosylation. Nat Commun. 2017;8(1):447 10.1038/s41467-017-00453-3 28878392 PMC5587582

[19] Zhao W, Rasheed A, Tikkanen E, Lee JJ, Butterworth AS, Howson JM, et al Identification of new susceptibility loci for type 2 diabetes and shared etiological pathways with coronary heart disease. Nat Genet. 2017;49(10):1450–1457. 10.1038/ng.3943 28869590 PMC5844224

[20] Baselmans BM, Jansen R, Ip HF, van Dongen J, Abdellaoui A, van de Weijer MP, et al Multivariate genome-wide analyses of the well-being spectrum. Nat Genet. 2019;51(3):445–451. 10.1038/s41588-018-0320-8 30643256

[21] Nath AP, Ritchie SC, Grinberg NF, Tang HH, Huang QQ, Teo SM, et al Multivariate genome-wide association analysis of a cytokine network reveals variants with widespread immune, haematological, and cardiometabolic pleiotropy. Am J Hum Genet. 2019;105(6):1076–1090. 10.1016/j.ajhg.2019.10.001 31679650 PMC6904835

[22] Andreassen OA, Thompson WK, Schork AJ, Ripke S, Mattingsdal M, Kelsoe JR, et al Improved detection of common variants associated with schizophrenia and bipolar disorder using pleiotropy-informed conditional false discovery rate. PLoS Genet. 2013;9(4):e1003455 10.1371/journal.pgen.1003455 23637625 PMC3636100

[23] Chung D, Yang C, Li C, Gelernter J, Zhao H. GPA: a statistical approach to prioritizing GWAS results by integrating pleiotropy and annotation. PLoS Genet. 2014;10(11):e1004787 10.1371/journal.pgen.1004787 25393678 PMC4230845

[24] Liley J, Wallace C. A pleiotropy-informed Bayesian false discovery rate adapted to a shared control design finds new disease associations from GWAS summary statistics. PLoS Genet. 2015;11(2):e1004926 10.1371/journal.pgen.1004926 25658688 PMC4450050

[25] Ming J, Wang T, Yang C. LPM: a latent probit model to characterize the relationship among complex traits using summary statistics from multiple GWASs and functional annotations. Bioinformatics. 2020;36(8):2506–2514. 10.1093/bioinformatics/btz947 31860024

[26] Zhang Q, Feitosa M, Borecki IB. Estimating and testing pleiotropy of single genetic variant for two quantitative traits. Genet Epidemiol. 2014;38(6):523–530. 10.1002/gepi.21837 25044106 PMC4169079

[27] Schaid DJ, Tong X, Larrabee B, Kennedy RB, Poland GA, Sinnwell JP. Statistical methods for testing genetic pleiotropy. Genetics. 2016;204(2):483–497. 10.1534/genetics.116.189308 27527515 PMC5068841

[28] Lutz SM, Fingerlin TE, Hokanson JE, Lange C. A general approach to testing for pleiotropy with rare and common variants. Genet Epidemiol. 2017;41(2):163–170. 10.1002/gepi.22011 27900789 PMC5472207

[29] Schaid DJ, Tong X, Batzler A, Sinnwell JP, Qing J, Biernacka JM. Multivariate generalized linear model for genetic pleiotropy. Biostatistics. 2017;20(1):111–128. 10.1093/biostatistics/kxx067 PMC6296339 29267957

[30] Price AL, Patterson NJ, Plenge RM, Weinblatt ME, Shadick NA, Reich D. Principal components analysis corrects for stratification in genome-wide association studies. Nat Genet. 2006;38(8):904–909. 10.1038/ng1847 16862161

[31] Kang HM, Sul JH, Service SK, Zaitlen NA, Kong Sy, Freimer NB, et al Variance component model to account for sample structure in genome-wide association studies. Nat Genet. 2010;42(4):348–354. 10.1038/ng.548 20208533 PMC3092069

[32] Broadaway KA, Cutler DJ, Duncan R, Moore JL, Ware EB, Jhun MA, et al A statistical approach for testing cross-phenotype effects of rare variants. Am J Hum Genet. 2016;98(3):525–540. 10.1016/j.ajhg.2016.01.017 26942286 PMC4800053

[33] Berger RL. In: Panchapakesan S, Balakrishnan N, editors. Likelihood ratio tests and intersection-union tests. Boston, MA: Birkhäuser Boston; 1997 p. 225–237.

[34] MacKinnon DP, Lockwood CM, Hoffman JM, West SG, Sheets V. A comparison of methods to test mediation and other intervening variable effects. Psychol Methods. 2002;7(1):83–104. 10.1037/1082-989X.7.1.83 11928892 PMC2819363

[35] Sobel ME. Asymptotic confidence intervals for indirect effects in structural equation models. Sociol Methodol. 1982;13:290–312. 10.2307/270723

[36] Huang YT. Genome-wide analyses of sparse mediation effects under composite null hypotheses. Ann Appl Stat. 2019;13(1):60–84. 10.1214/18-AOAS1181

[37] Craig CC. On the frequency function of *xy*. Ann Math Statist. 1936;7(1):1–15.

[38] R Core Team. R: A language and environment for statistical computing; 2018. Available from: https://www.R-project.org/.

[39] MacKinnon DP, Warsi G, Dwyer JH. A simulation study of mediated effect measures. Multivariate Behav Res. 1995;30(1):41–62. 10.1207/s15327906mbr3001_3 20157641 PMC2821114

[40] Wellcome Trust Case Control Consortium, et al Genome-wide association study of 14,000 cases of seven common diseases and 3,000 shared controls. Nature. 2007;447(7145):661–678. 10.1038/nature05911 17554300 PMC2719288

[41] Mitchell BD, Fornage M, McArdle PF, Cheng YC, Pulit S, Wong Q, et al Using previously genotyped controls in genome-wide association studies (GWAS): application to the Stroke Genetics Network (SiGN). Front Genet. 2014;5:95 10.3389/fgene.2014.00095 24808905 PMC4010766

[42] Lin DY, Sullivan PF. Meta-analysis of genome-wide association studies with overlapping subjects. Am J Hum Genet. 2009;85(6):862–872. 10.1016/j.ajhg.2009.11.001 20004761 PMC2790578

[43] Ray D, Boehnke M. Methods for meta-analysis of multiple traits using GWAS summary statistics. Genet Epidemiol. 2018;42(2):134–145. 10.1002/gepi.22105 29226385 PMC5811402

[44] Bulik-Sullivan B, Finucane HK, Anttila V, Gusev A, Day FR, Loh PR, et al An atlas of genetic correlations across human diseases and traits. Nat Genet. 2015;47(11):1236–1241. 10.1038/ng.3406 26414676 PMC4797329

[45] Wang T, Elston RC. Improved power by use of a weighted score test for linkage disequilibrium mapping. Am J Hum Genet. 2007;80(2):353–360. 10.1086/511312 17236140 PMC1785334

[46] Basu S, Pan W. Comparison of statistical tests for disease association with rare variants. Genet Epidemiol. 2011;35(7):606–619. 10.1002/gepi.20609 21769936 PMC3197766

[47] Ray D, Li X, Pan W, Pankow JS, Basu S. A Bayesian partitioning model for the detection of multilocus effects in case-control studies. Hum Hered. 2015;79(2):69–79. 10.1159/000369858 26044550 PMC4499013

[48] Giovannucci E, Rimm EB, Stampfer MJ, Colditz GA, Willett WC. Diabetes mellitus and risk of prostate cancer (United States). Cancer Causes Control. 1998;9(1):3–9. 10.1023/A:1008822917449 9486458

[49] Kasper JS, Giovannucci E. A meta-analysis of diabetes mellitus and the risk of prostate cancer. Cancer Epidemiol Biomarkers Prev. 2006;15(11):2056–2062. 10.1158/1055-9965.EPI-06-0410 17119028

[50] Waters KM, Henderson BE, Stram DO, Wan P, Kolonel LN, Haiman CA. Association of diabetes with prostate cancer risk in the multiethnic cohort. Am J Epidemiol. 2009;169(8):937–945. 10.1093/aje/kwp003 19240222 PMC2727229

[51] Machiela MJ, Lindström S, Allen NE, Haiman CA, Albanes D, Barricarte A, et al Association of type 2 diabetes susceptibility variants with advanced prostate cancer risk in the Breast and Prostate Cancer Cohort Consortium. Am J Epidemiol. 2012;176(12):1121–1129. 10.1093/aje/kws191 23193118 PMC3571230

[52] Gallagher EJ, LeRoith D. Epidemiology and molecular mechanisms tying obesity, diabetes, and the metabolic syndrome with cancer. Diabetes Care. 2013;36(Supplement 2):S233–S239. 10.2337/dcS13-2001 23882051 PMC3920794

[53] Frayling T, Colhoun H, Florez J. A genetic link between type 2 diabetes and prostate cancer. Diabetologia. 2008;51(10):1757–1760. 10.1007/s00125-008-1114-9 18696045

[54] Pierce BL, Ahsan H. Genetic susceptibility to type 2 diabetes is associated with reduced prostate cancer risk. Hum Hered. 2010;69(3):193–201. 10.1159/000289594 20203524 PMC2866577

[55] Meyer TE, Boerwinkle E, Morrison AC, Volcik KA, Sanderson M, Coker AL, et al Diabetes genes and prostate cancer in the Atherosclerosis Risk in Communities study. Cancer Epidemiol Biomarkers Prev. 2010;19(2):558–565. 10.1158/1055-9965.EPI-09-0902 20142250 PMC2820124

[56] Yu OHY, Foulkes WD, Dastani Z, Martin RM, Eeles R, Richards JB, et al An assessment of the shared allelic architecture between type II diabetes and prostate cancer. Cancer Epidemiol Biomarkers Prev. 2013;22(8):1473–1475. 10.1158/1055-9965.EPI-13-0476 23704474 PMC4158605

[57] Xue A, Wu Y, Zhu Z, Zhang F, Kemper KE, Zheng Z, et al Genome-wide association analyses identify 143 risk variants and putative regulatory mechanisms for type 2 diabetes. Nat Commun. 2018;9(1):2941 10.1038/s41467-018-04951-w 30054458 PMC6063971

[58] Morris AP, Voight BF, Teslovich TM, Ferreira T, Segre AV, Steinthorsdottir V, et al Large-scale association analysis provides insights into the genetic architecture and pathophysiology of type 2 diabetes. Nat Genet. 2012;44(9):981–990. 10.1038/ng.2383 22885922 PMC3442244

[59] Banda Y, Kvale MN, Hoffmann TJ, Hesselson SE, Ranatunga D, Tang H, et al Characterizing race/ethnicity and genetic ancestry for 100,000 subjects in the Genetic Epidemiology Research on Adult Health and Aging (GERA) cohort. Genetics. 2015;200(4):1285–1295. 10.1534/genetics.115.178616 26092716 PMC4574246

[60] Bycroft C, Freeman C, Petkova D, Band G, Elliott LT, Sharp K, et al The UK Biobank resource with deep phenotyping and genomic data. Nature. 2018;562(7726):203–209. 10.1038/s41586-018-0579-z 30305743 PMC6786975

[61] Schumacher FR, Al Olama AA, Berndt SI, Benlloch S, Ahmed M, Saunders EJ, et al Association analyses of more than 140,000 men identify 63 new prostate cancer susceptibility loci. Nat Genet. 2018;50(7):928 10.1038/s41588-018-0142-8 29892016 PMC6568012

[62] Bulik-Sullivan BK, Loh PR, Finucane HK, Ripke S, Yang J, Patterson N, et al LD Score regression distinguishes confounding from polygenicity in genome-wide association studies. Nat Genet. 2015;47(3):291–295. 10.1038/ng.3211 25642630 PMC4495769

[63] Zheng J, Erzurumluoglu AM, Elsworth BL, Kemp JP, Howe L, Haycock PC, et al LD Hub: a centralized database and web interface to perform LD score regression that maximizes the potential of summary level GWAS data for SNP heritability and genetic correlation analysis. Bioinformatics. 2016;33(2):272–279. 10.1093/bioinformatics/btw613 27663502 PMC5542030

[64] Watanabe K, Taskesen E, Van Bochoven A, Posthuma D. Functional mapping and annotation of genetic associations with FUMA. Nat Commun. 2017;8(1):1826 10.1038/s41467-017-01261-5 29184056 PMC5705698

[65] Giambartolomei C, Vukcevic D, Schadt EE, Franke L, Hingorani AD, Wallace C, et al Bayesian test for colocalisation between pairs of genetic association studies using summary statistics. PLoS Genet. 2014;10(5):e1004383 10.1371/journal.pgen.1004383 24830394 PMC4022491

[66] Guo H, Fortune MD, Burren OS, Schofield E, Todd JA, Wallace C. Integration of disease association and eQTL data using a Bayesian colocalisation approach highlights six candidate causal genes in immune-mediated diseases. Hum Mol Genet. 2015;24(12):3305–3313. 10.1093/hmg/ddv077 25743184 PMC4498151

[67] Watanabe K, Stringer S, Frei O, Mirkov MU, de Leeuw C, Polderman TJ, et al A global overview of pleiotropy and genetic architecture in complex traits. Nat Genet. 2019;51(9):1339–1348. 10.1038/s41588-019-0481-0 31427789

[68] Koscielny G, An P, Carvalho-Silva D, Cham JA, Fumis L, Gasparyan R, et al Open Targets: a platform for therapeutic target identification and validation. Nucleic Acids Res. 2016;45(D1):D985–D994. 10.1093/nar/gkw1055 27899665 PMC5210543

[69] Võsa U, Claringbould A, Westra HJ, Bonder MJ, Deelen P, Zeng B, et al. Unraveling the polygenic architecture of complex traits using blood eQTL metaanalysis. bioRxiv. 2018

[70] Mahajan A, Taliun D, Thurner M, Robertson NR, Torres JM, Rayner NW, et al Fine-mapping type 2 diabetes loci to single-variant resolution using high-density imputation and islet-specific epigenome maps. Nat Genet. 2018;50:1505–1513. 10.1038/s41588-018-0241-6 30297969 PMC6287706

[71] GTEx Consortium, et al Genetic effects on gene expression across human tissues. Nature. 2017;550(7675):204–213. 10.1038/nature24277 29022597 PMC5776756

[72] Benjamini Y, Hochberg Y. Controlling the false discovery rate: a practical and powerful approach to multiple testing. J R Statist Soc B. 1995;57(1):289–300.

[73] Bonovas S, Filioussi K, Tsantes A. Diabetes mellitus and risk of prostate cancer: a meta-analysis. Diabetologia. 2004;47(6):1071–1078. 10.1007/s00125-004-1415-6 15164171

[74] Tande AJ, Platz EA, Folsom AR. The metabolic syndrome is associated with reduced risk of prostate cancer. Am J Epidemiol. 2006;164(11):1094–1102. 10.1093/aje/kwj320 16968859

[75] Buniello A, MacArthur JAL, Cerezo M, Harris LW, Hayhurst J, Malangone C, et al The NHGRI-EBI GWAS Catalog of published genome-wide association studies, targeted arrays and summary statistics 2019. Nucleic Acids Res. 2019;47(D1):D1005–D1012. 10.1093/nar/gky1120 30445434 PMC6323933

[76] Bioconductor Package Maintainer. liftOver: Changing genomic coordinate systems with rtracklayer::liftOver.; 2019. Available from: https://www.bioconductor.org/help/workflows/liftOver/.

[77] Gudmundsson J, Sulem P, Steinthorsdottir V, Bergthorsson JT, Thorleifsson G, Manolescu A, et al Two variants on chromosome 17 confer prostate cancer risk, and the one in *TCF2* protects against type 2 diabetes. Nat Genet. 2007;39(8):977–983. 10.1038/ng2062 17603485

[78] Liberzon A, Subramanian A, Pinchback R, Thorvaldsdóttir H, Tamayo P, Mesirov JP. Molecular signatures database (MSigDB) 3.0. Bioinformatics. 2011;27(12):1739–1740. 10.1093/bioinformatics/btr260 21546393 PMC3106198

[79] Kutmon M, Riutta A, Nunes N, Hanspers K, Willighagen EL, Bohler A, et al WikiPathways: capturing the full diversity of pathway knowledge. Nucleic Acids Res. 2015;44(D1):D488–D494. 10.1093/nar/gkv1024 26481357 PMC4702772

[80] Lindström S, Finucane H, Bulik-Sullivan B, Schumacher FR, Amos CI, Hung RJ, et al Quantifying the genetic correlation between multiple cancer types. Cancer Epidemiol Biomarkers Prev. 2017;26(9):1427–1435. 10.1158/1055-9965.EPI-17-0211 28637796 PMC5582139

