# Supplementary material for: A powerful method for pleiotropic analysis under composite null hypothesis identifies novel shared loci between Type 2 Diabetes and Prostate Cancer
Source: PLoS Genet. 2020 Dec 8;16(12):e1009218. doi: 10.1371/journal.pgen.1009218 (PMC7748289; doi:10.1371/journal.pgen.1009218)
Supplement: S1 Appendix — It includes additional details on PLACO p-value calculation, simulation experiments, and analysis of T2D and PrCa datasets. (PDF) [file pgen.1009218.s001.pdf]

S1 Appendix

Section A

Calculation of PLACO (approximate) p-value. The analytical p-value (two-tailed) for testing $H_0 : \beta_1\beta_2 = 0$ (no pleiotropy) against $H_a : \beta_1\beta_2 \neq 0$ using the product of Z-scores as our PLACO test statistic is given by

$$p_{Z_1Z_2} = \pi_{00}\mathbb{F}(z_1z_2) + \pi_{01}\mathbb{F}\left(z_1z_2/\sqrt{1+\tau_2^2}\right) + \pi_{02}\mathbb{F}\left(z_1z_2/\sqrt{1+\tau_1^2}\right) \qquad \text{(Eq 2)}$$

which involves the mixture probabilities π_{00}, π_{01} and π_{02} for the sub-null hypotheses H_{00}, H_{01} and H_{02} to hold respectively. Accurately estimating these mixture probabilities under different scenarios is difficult. Instead, we used an asymptotic approximate form of this p-value[1]

$$\hat{p}_{Z_1Z_2} = \mathbb{F}\left(z_1z_2/\sqrt{\text{Var}(Z_1)}\right) + \mathbb{F}\left(z_1z_2/\sqrt{\text{Var}(Z_2)}\right) - \mathbb{F}\left(z_1z_2\right) \qquad \text{(Eq 3)}$$

which involves only two of the five unknown parameters through the marginal variances $\text{Var}(Z_1) = 1+\pi_{02}\tau_1^2$ and $\text{Var}(Z_2) = 1+\pi_{01}\tau_2^2$. These are the variances of the single-trait Z-scores (Wald test statistics) Z_1 and Z_2 respectively under the composite null H_0. We estimate $\text{Var}(Z_1)$ and $\text{Var}(Z_2)$ using the millions of "null" genetic variants genome-wide. *A priori* it is not known which variants are "null" (i.e., satisfy the composite null of no pleiotropy). So, we choose a significance threshold, say 10^{-4}, for the single-trait p-values using which we decide whether a variant is under sub-null H_{01} or H_{02} or H_{00}. This way, we collect all the variants satisfying any of the three sub-nulls and calculate sample variance of the Z-scores from these variants. This estimation procedure is done only once for a given study using the single-trait Z-scores and p-values (or GWAS summary statistics) that are usually publicly available.

Now, the choice of this significance threshold to declare a variant "null" or "non-null" for a given trait is arbitrary. We evaluated sensitivity of this threshold choice on the type I error control and power of PLACO using the simulated data under Scenarios I and II (as described in the main manuscript). In particular, we used thresholds of $10^{-2}, 10^{-3}, 10^{-4}$ and 5×10^{-5}, and found PLACO's type I error control (**Fig A5**) and power (**Table A2**; Scenario I only) qualitatively similar across different choices of threshold.

Note, this estimation procedure is similar to how trait correlation matrix is estimated for multi-trait analysis using GWAS summary statistics[2;3]. Ideally, nearly independent variants across the genome should be used for calculating these estimates; however, not filtering out dependent variants in this genome-wide estimation process is not expected to have any critical impact on the overall behavior of such methods[2]. This is also evident from our sensitivity analyses that slightly varying estimates of parameters involved in the PLACO p-value calculation does not affect PLACO's type I error control. For instance, the above sensitivity analysis on choice of threshold to define "null" variants - although different choices may lead to slightly different parameter estimates, the overall behavior of PLACO appears to be robust (**Fig A5**). Another example is how the different ways of estimating study correlations (Lin-Sullivan[4] vs Pearson correlation approach) may lead to slightly different correlation estimates, yet we found PLACO's performance to be qualitatively similar (figures not shown).

Estimation of PLACO analytical p-value. The analytical form for PLACO p-value in Eq 2 contains unknown parameters π_{00}, π_{01}, π_{02}, τ_1 and τ_2. To estimate these parameters, one may employ the approach taken by cross-phenotype summary-statistics based methods to estimate the covariance matrix of multiple Z-scores[3]. For instance, the proportion of genetic variants with marginal p-values $> 10^{-4}$ for both traits can be used as an estimate of π_{00}. An estimate of π_{01} is the proportion of genetic variants with p-values $> 10^{-4}$ for the first trait and with p-values $< 10^{-4}$ for the second trait. π_{02} can be similarly estimated. For estimating τ_1, observe that the variance of Z_1 under H_{02} is $1 + \tau_1^2$. Therefore, the estimated variance of Z_1 corresponding to genetic variants with p-values $< 10^{-4}$ for the first trait and with p-values $> 10^{-4}$ for the second trait can be used as an estimate of $1 + \tau_1^2$ in Eq 2. Similarly, the variance parameter τ_2 can be estimated. Note that this estimation procedure (denoted as 'PLACO (estimated pvalue)' in **Fig A1**) is done only once for a given study using the single-trait Z-scores and p-values ('summary statistics') that are usually publicly available from a GWAS. We found this p-value calculation approach conservative for stringent significance levels (**Fig A1**), and hence use the approximate p-value calculation (Eq 3) for PLACO everywhere in this manuscript. The `PLACO` software, too, reports the approximate p-value.

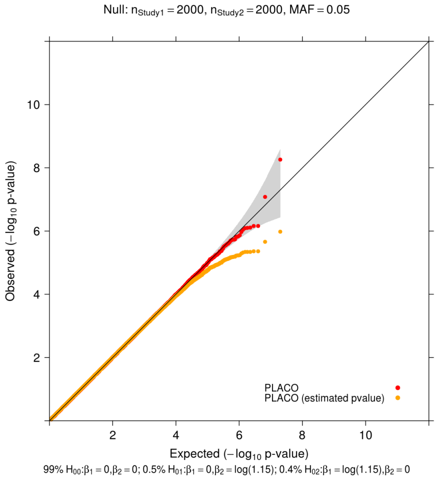

(a) 1:1 study sample size

Fig A1: Scenario I: Comparison of the PLACO approach using the asymptotic approximate p-value vs using the estimated p-value. QQ plots for null data are plotted on 2 case-control traits from 2 independent studies with fixed genetic effects. Observed($-\log_{10}$p-values) are plotted on the y-axis and Expected($-\log_{10}$p-values) on the x-axis. Each study has $1,000$ unrelated cases and $1,000$ unrelated controls. Performance of the tests of pleiotropic effect of a genetic variant on the 2 traits is based on 9.99 million variants. The gray shaded region represents a conservative 95% confidence interval for the expected distribution of p-values.

Section B

Correlation between case-control studies with shared controls. For two outcomes from two case-control studies, the correlation between the Z-scores is

$$\rho \approx \left(n_{12,\text{co}} \sqrt{\frac{n_{1,\text{ca}} n_{2,\text{ca}}}{n_{1,\text{co}} n_{2,\text{co}}}} + n_{12,\text{ca}} \sqrt{\frac{n_{1,\text{co}} n_{2,\text{co}}}{n_{1,\text{ca}} n_{2,\text{ca}}}} \right) / \sqrt{n_1 n_2}$$

(ignoring the variation due to $\hat{\text{se}}(\hat{\beta}_k)$'s) under the global null of no association, where $n_{k,\text{ca}}$ and $n_{k,\text{co}}$ are respectively the number of cases and the number of controls in the study for k-th outcome, and $n_{12,\text{co}}$ ($n_{12,\text{ca}}$) is the number of shared controls (cases) between the two studies[4]. In reality, the cases in two case-control studies are always independent and the control group in each study is at least as large as the case group. Based on this, let us assume (1) 100% control overlap and no shared case ($n_{12,\text{ca}} = 0$); (2) the case:control ratio in both studies is the same (say, 1:r_c, where $r_c \geq 1$ – a reasonable assumption because the number of controls is almost always larger than the number of cases); (3) the total sample size of study 1 is r_s times that of study 2, where $r_s \geq 1$. From assumption (1), the correlation boils down to $\rho \approx \left(n_{12,\text{co}} \sqrt{\frac{n_{1,\text{ca}} n_{2,\text{ca}}}{n_{1,\text{co}} n_{2,\text{co}}}} \right) / \sqrt{n_1 n_2}$. From assumptions (1) and (2), we get $n_{12,\text{co}} = n_{1,\text{co}} = n_{2,\text{co}}$ and $n_{j,\text{co}} = r_c n_{j,\text{ca}}$ for study $j = 1, 2$. From assumptions (2) and (3), we have $n_1 = r_s n_2$, $n_{1,\text{ca}} = r_s n_{2,\text{ca}}$, and $n_{1,\text{co}} = r_s n_{2,\text{co}}$. Finally, under these 3 assumptions, we have $\rho \approx \frac{1}{\sqrt{r_s}(1+r_c)}$, the maximum of which is attained when r_s and r_c take the lowest possible value. Thus, the correlation ρ reaches a maximum of 0.5 when there are equal numbers of cases and controls in each study, both studies have the same sample size and all the controls (and no case) are shared.

Table A1: Possible values of correlation ρ between Z-scores of two outcomes from two case-control studies with complete control overlap, with one study being $r_s(\geq 1)$ times as large as the other study, and with $r_c(\geq 1)$ controls for each case in both studies.

1:r_c \ r_s:1	1:1	1.5:1	2:1	3:1	5:1	10:1
1:1	0.5	0.41	0.35	0.29	0.22	0.16
1:4	0.2	0.16	0.14	0.11	0.09	0.06
1:9	0.1	0.08	0.07	0.06	0.04	0.03

Section C

Generative model for simulation experiments. For each individual, we first generate the genotype of a variant by simulating a latent normal model with mean 0 and variance 1. We dichotomize the latent normal variable into a haplotype based on whether it is smaller than the MAF of the variant or not. Similarly we generate another independent haplotype, which is then combined with the previous haplotype to obtain a genotype for the indivdual. Next, for a given disease trait in Scenario I or II, we assume the simulated variant is causal and use a main effects logistic model for the probability that an individual has the disease to generate the disease status. We repeat this process until we have the required number of cases and controls for both traits. We emphasize that this generative model for our simulated data have been widely used before[5–7], and is distinct from the hierarchical model assumed by PLACO. For Scenario III, the generative model is the same as before except that a bivariate normal model with means 0, variances 1, and pairwise correlation ρ_{trait} is used to simulate the quantitative traits.

Type I error performance under more general simulation settings. Here we evaluate sensitivity (if any) of type I error control of PLACO for simulation settings not considered in the original composite null simulations presented in the main manuscript under Scenarios I (independent case-control studies), II (case-control studies with overlapping controls) and/or III (correlated quantitative traits). Unless otherwise mentioned, other simulation settings are the same as described in the main manuscript.

Weaker genetic effect of the associated trait under the null. One reviewer pointed out that the type I error rates may not be well-controlled when odds ratio (OR) for the associated trait is very close to 1. In our original null simulation settings, we assume 99% of the 10 million variants to be under the global null $H_{00} : \mathrm{OR}_1 = 1, \mathrm{OR}_2 = 1$; 0.5% variants under the sub-null $H_{01} : \mathrm{OR}_1 = 1, \mathrm{OR}_2 = 1.15$ and 0.4% variants under the sub-null $H_{02} : \mathrm{OR}_1 = 1.15, \mathrm{OR}_2 = 1$. Here, we instead consider a smaller associated OR of 1.05 for Scenario I, and still observe well-controlled type I error (**Fig A2**).

Higher proportion of null SNPs not under the global null, and varying MAF. To evaluate sensitivity of PLACO when higher proportions of null (i.e., non-pleiotropic) variants with weak effect

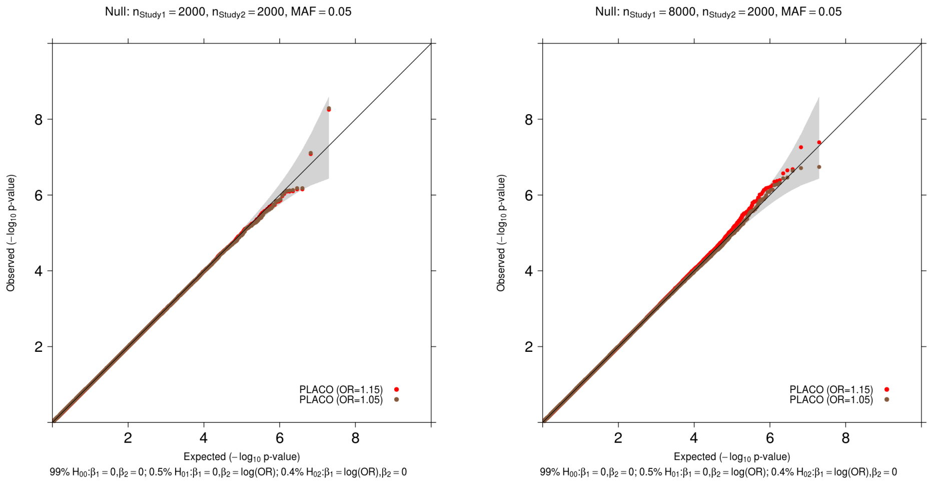

(a) 1:1 study sample size (b) 4:1 study sample size

Fig A2: Scenario I: Comparison of the PLACO approach for different fixed values of the associated trait effect under the null for traits from 2 independent case-control studies. Observed($-\log_{10}$p-values) are plotted on the y-axis and Expected($-\log_{10}$p-values) on the x-axis. Either each study has $1,000$ unrelated cases and $1,000$ unrelated controls, or Study 1 is 4 times that of Study 2, where Study 2 has $1,000$ unrelated cases and $1,000$ unrelated controls. Type I error performance is based on 9.99 million null variants with genetic effects that are either $\{\beta_1 = 0 = \beta_2\}$ or $\{\beta_1 = 0, \beta_2 = \log(\text{OR})\}$ or $\{\beta_1 = \log(\text{OR}), \beta_2 = 0\}$, where OR is either 1.15 (as in the original simulation settings) or weaker at 1.05. The gray shaded region represents a conservative 95% confidence interval for the expected distribution of p-values.

sizes for one trait are observed, we consider a more general situation where a distribution is assumed for one of the genetic effects.

For Scenario I, we use a normal distribution with mean 0 and standard deviation 0.1 for the genetic effect of the first trait (the choice of this distribution is motivated by the distribution of effect sizes of common variants across many complex human traits[8]). The genetic effect of the second trait is fixed at 0 (or odds ratio = 1). In other words, out of the 10 million genetic variants, we assume 99.9% variants to be under either the global null H_{00} (i.e., none of the traits is associated) or the sub-null H_{02} (i.e., only first trait is associated). Note, the way we have simulated here, the probability of a variant to be under the global null $H_{00} : \text{OR}_1 = 1, \text{OR}_2 = 1$ is very small, and thus the majority of the null variants is expected to be under the sub-null $H_{02} : \text{OR}_1 \neq 1, \text{OR}_2 = 1$. For the 0.1% non-null variants, we assume the same distribution for the first genetic effect and fix the genetic effect for the second trait at an arbitrary non-null value. To additionally study how PLACO and other methods perform across different values of MAF, we not only consider MAF 5% but also consider MAF of 10%, 20%, and 40%. We observe that regardless of the MAF or the skewness in sample sizes of traits or the proportion of variants under

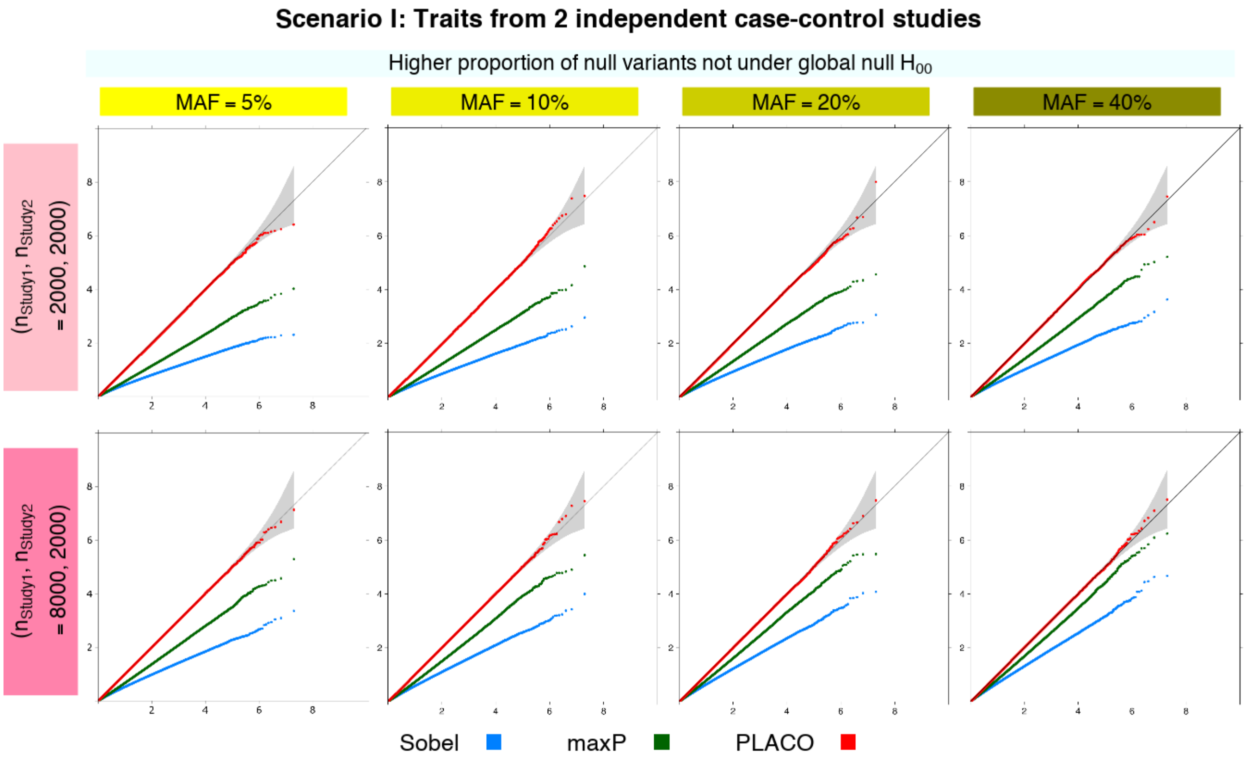

Fig A3: Scenario I: QQ plots for the pleiotropic analysis of null data on traits from 2 independent case-control studies with distribution assumed for genetic effect of 1 trait. Observed($-\log_{10}$p-values) are plotted on the y-axis and Expected($-\log_{10}$p-values) on the x-axis. Either each study has $1,000$ unrelated cases and $1,000$ unrelated controls, or Study 1 has 4 times sample size as Study 2, where Study 2 has $1,000$ unrelated cases and $1,000$ unrelated controls. Type I error performance of tests of pleiotropic effect of a genetic variant on the 2 traits is based on 9.99 million null variants with genetic effects that are $\{\beta_1 \sim N(0, 0.1^2), \beta_2 = 0\}$. Thus, majority of the null variants are under the sub-null H_{02} unlike the original simulation settings where a large fixed proportion of null variants are under the global null H_{00}. The gray shaded region represents a conservative 95% confidence interval for the expected distribution of p-values.

the global null, PLACO seems to have good type I error control (**Fig A3**).

We further evaluated this effect of not having a fixed major proportion of null variants under global null for Scenario II. This time we only focused on MAF 5% and the setting with equal sample sizes for both traits, and again observed well-controlled type I error for PLACO across varying levels of control overlap once the overlap is accounted for (**Fig A4**).

Sensitivity analysis: choice of threshold to define "null" variants for calculating PLACO p-value. As described in Section A, calculating approximate p-value of PLACO requires estimating $\text{Var}(Z_1)$ and $\text{Var}(Z_2)$ based on the millions of "null" variants genome-wide. Here we explore if the choice of 'arbitrary' significance threshold (default choice in the `PLACO` software is 10^{-4}) used to declare a variant "null" or "non-null" for a given trait affects the type I error control and power of PLACO.

For type I error evaluation, we simulate null data under Scenarios I and II as described in the

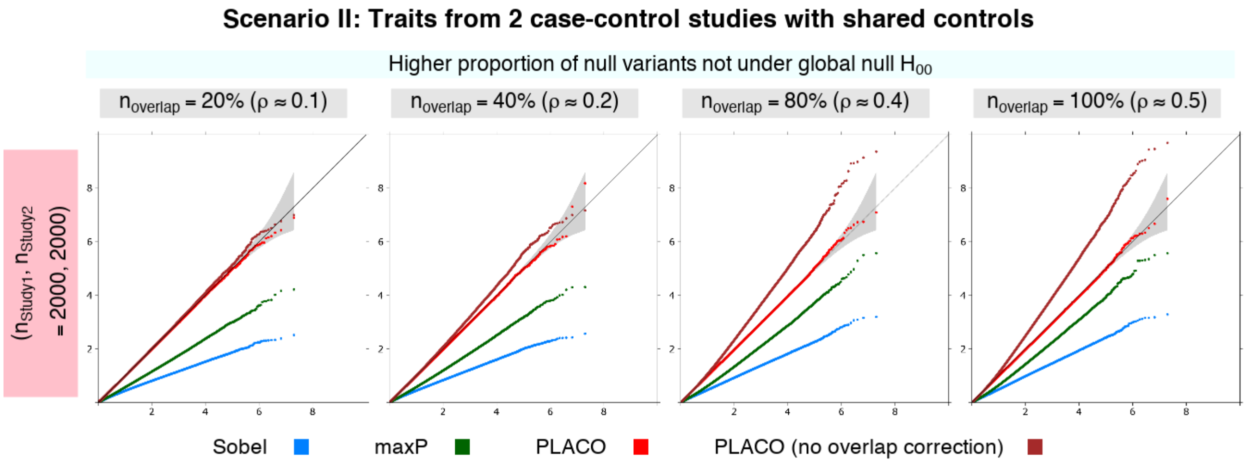

Fig A4: Scenario II: QQ plots for the pleiotropic analysis of null data on traits from 2 case-control studies with different proportions of overlapping controls and with distribution assumed for genetic effect of 1 trait. Observed($-\log_{10}$p-values) are plotted on the y-axis and Expected($-\log_{10}$p-values) on the x-axis. Equal study sample size, and equal case-control size assumed in each study. Each study has $1,000$ unrelated cases and $1,000$ unrelated controls, of which either 20%, 40%, 80% or 100% of the controls are shared between the two studies. Type I error performance of tests of pleiotropic effect of a genetic variant on the 2 traits is based on 9.99 million null variants with genetic effects that are $\{\beta_1 \sim N(0, 0.1^2),\ \beta_2 = 0\}$. Thus, majority of the null variants are under the sub-null H_{02} unlike the original simulation settings where a large fixed proportion of null variants are under the global null H_{00}. The gray shaded region represents a conservative 95% confidence interval for the expected distribution of p-values.

main manuscript. In particular, out of the 9.99 million independent null variants, we assume 9.90 million variants are under the global null ($\text{OR}_1 = 1, \text{OR}_2 = 1$), 50 thousand variants influence risk of Trait 2 only ($\text{OR}_1 = 1, \text{OR}_2 = 1.15$) and 40 thousand variants influence risk of Trait 1 only ($\text{OR}_1 = 1.15, \text{OR}_2 = 1$). Additionally, we consider a separate simulation setting where, for all 9.99 million null variants, we assume a normal distribution with mean 0 and standard deviation 0.1 for $\log(\text{OR}_1)$ and fix $\text{OR}_2 = 1$. This setting covers a scenario where most null (non-pleiotropic) variants are not under the global null since higher proportions of associated variants with weak effect sizes are often observed for many complex traits. For power evaluation, we simulate 10 thousand independent pleiotropic variants with different choices of OR_1 and OR_2 under Scenario I only. Evaluating power for Scenario II is redundant since power depends on the total number of independent subjects, which we explore in Scenario I.

When applying PLACO on these simulated data, we calculate $\text{Var}(Z_1)$ and $\text{Var}(Z_2)$ only once on the entire combination of 10 million independent variants. Assuming we do not know which variants are "null", we separately use thresholds of 10^{-2}, 10^{-3}, 10^{-4} or 5×10^{-5} to classify variants as "null" or "non-null". We found PLACO's type I error control (**Fig A5**) and power (**Table**

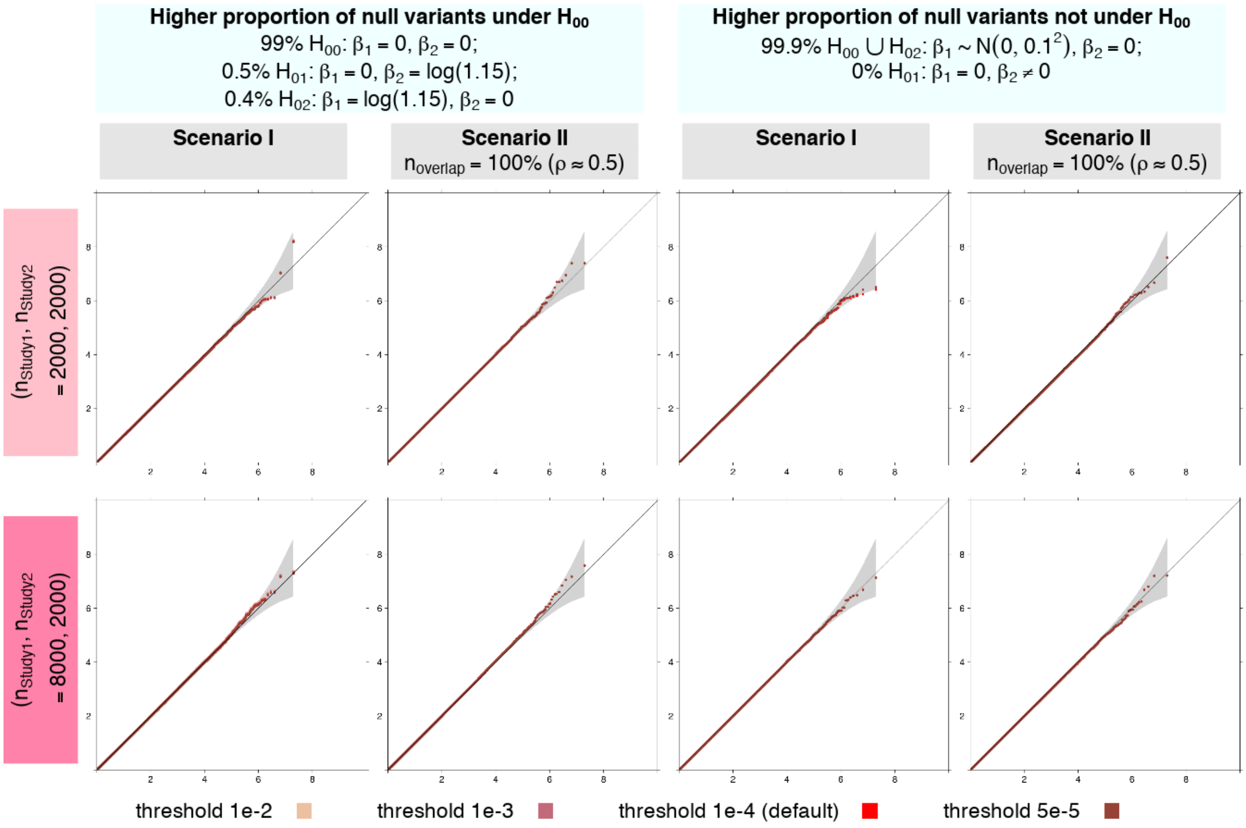

Fig A5: Scenarios I & II: Comparison of PLACO type I error control across different choices of threshold used to define "null" variants for estimating parameters needed in PLACO's p-value calculation. Observed($-\log_{10}$p-values) are plotted on the y-axis and Expected($-\log_{10}$p-values) on the x-axis. Either each study has 1,000 unrelated cases and 1,000 unrelated controls, or Study 1 has 4 times sample size as Study 2, where Study 2 has 1,000 unrelated cases and 1,000 unrelated controls. For Scenario II, we focus on the extreme case with 100% control overlap. The gray shaded region represents a conservative 95% confidence interval for the expected distribution of p-values.

A2; Scenario I only) qualitatively similar across different choices of this threshold.

Table A2: Scenario I: Sensitivity of PLACO's power across different choices of threshold used to define "null" variants for estimating parameters needed in PLACO's p-value calculation. Power (reported here in %) at genome-wide significance level (5×10^{-8}) is based on 10,000 simulated pleiotropic variants with effect sizes OR_1 and OR_2 on the two case-control traits respectively. Either each study has 1,000 unrelated cases and 1,000 unrelated controls, or Study 1 has 4 times sample size as Study 2, where Study 2 has 1,000 unrelated cases and 1,000 unrelated controls.

Effect size	Study size	Threshold			
		10^{-2}	10^{-3}	10^{-4}	5×10^{-5}
$OR_1 = 1.5, OR_2 = 1.5$	1:1	7.3	7.1	7.1	7.1
	4:1	60.8	60.6	60.0	60.0
$OR_1 = 1.7, OR_2 = 1.7$	1:1	48.2	47.8	47.4	47.2
	4:1	97.3	97.2	97.1	97.1
$OR_1 = 1.5, OR_2 = \frac{1}{1.5}$	1:1	4.8	4.8	4.8	4.8
	4:1	46.6	46.0	45.7	45.7
$OR_1 = 1.7, OR_2 = \frac{1}{1.7}$	1:1	28.9	28.4	28.1	28.1
	4:1	89.9	89.6	89.3	89.2

Section D

More on gene-set enrichment analysis. `FUMA` also performed enrichment analyses in other annotated gene sets described in Molecular Signatures Database (MSigDB v7.0)[9] and in curated biological pathways from WikiPathways[10]. We found significant enrichment in 3 gene sets representing expression signatures of genetic and chemical perturbations (**Fig A6**); in 2 gene sets representing potential targets of regulation by transcription factors (**Fig A7**); and in 1 gene set representing cell states and perturbations within the immune system (**Fig A8**). Delving deeper into these gene sets and pathways may provide knowledge about T2D-PrCa etiology and may shed light on the observed T2D-PrCa inverse association[11]; however delving deeper is beyond the scope of this article.

Functional enrichment analysis. We tested enrichment of functional consequences of 43 loci using `FUMA` (we excluded the MHC locus from all analyses because of strong SNP associations in this long-range and complex LD block that complicates fine-mapping efforts[12]). Fisher's exact tests of enrichment for 11 annotations show significant enrichment of SNPs in flanking regions such as 3-prime ($p_{\text{Fisher}} = 2.4 \times 10^{-23}$) and 5-prime UTRs ($p_{\text{Fisher}} = 7.2 \times 10^{-5}$) (**Table A3**). We found 46 (3.6%) significant SNPs spread across 19 loci have CADD scores[13] > 12.37 (the suggested threshold for deleteriousness, as reported in the `FUMA` documentation). RegulomeDB categorical scores[14] predict 35 SNPs across 9 loci to affect binding and linked to expression of a gene target, while 33 SNPs across 18 loci are likely to affect binding only. Majority of the significant SNPs has highly significant *cis*-regulatory effects ($p < 5 \times 10^{-8}$) on gene expression in whole blood from eQTLGen Consortium.

Colocalization analysis. Bayesian colocalization tests of ±200 Kb region around the lead SNPs of the 43 loci reveal 26 lead SNPs as having the highest posterior probability of being associated with both PrCa and T2D. Interestingly, the pleiotropic loci near known shared genes *THADA* and *JAZF1*, near candidate shared genes *PPARG* and *CDKN2A*, and near previously implicated GWAS catalog genes *BCL2L11* and *AC005355.2* do not have convincing evidence of being driven by a single causal variant for both diseases. Pleiotropic signals at these loci are likely driven by two distinct causal SNPs for the two diseases in strong LD with each other.

Table A3: Enrichment statistics for different functional consequences (annotations from ANNOVAR) of SNPs in LD with the lead SNPs from all 43 loci detected by PLACO. 1000G Phase 3 European population is used as reference panel.

Annotation	Count (ref.)	Prop. (ref.)	Count (here)	Prop. (here)	Enrichment	p_{Fisher}
UTR3	233824	0.00932	211	0.020	2.150	2.4×10^{-23}
UTR5	71546	0.00285	54	0.005	1.798	7.2×10^{-5}
downstream	284177	0.01133	132	0.013	1.107	2.5×10^{-1}
exonic	254736	0.01016	131	0.012	1.225	2.2×10^{-2}
intergenic	11684523	0.46586	2252	0.214	0.459	0
intronic	9137749	0.36432	6308	0.599	1.645	0
ncRNA_exonic	259951	0.01036	106	0.010	0.972	8.1×10^{-1}
ncRNA_intronic	2884355	0.11500	1211	0.115	1.000	9.9×10^{-1}
ncRNA_splicing	1313	0.00005	0	0.000	0.000	1
splicing	2830	0.00011	0	0.000	0.000	6.4×10^{-1}
upstream	266686	0.01063	121	0.011	1.081	3.9×10^{-1}

Annotation: Functional consequence of SNPs
Count (ref.): Number of SNPs with the corresponding annotation in the reference panel
Prop. (ref.): Proportion of SNPs with the corresponding annotation in the reference panel
Count (here): Number of SNPs with the corresponding annotation in the candidate set of SNPs from current GWAS
Prop. (here): Proportion of SNPs with the corresponding annotation in the candidate set of SNPs from current GWAS
Enrichment: Ratio of prop. (here) to prop. (ref.); value > 1 indicates annotation is enriched, otherwise depleted
p_{Fisher} : p-value from Fisher's exact test (2-sided test)

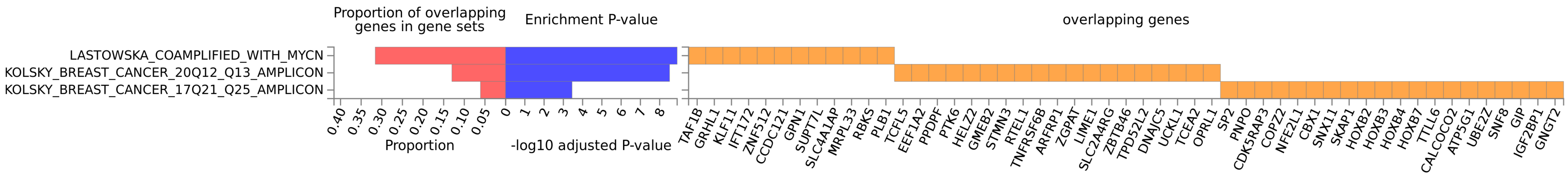

Fig A6: **Mapped genes (as done by** FUMA**) for the** 43 **pleiotropic loci detected by PLACO were tested for enrichment in MsigDB C2 gene sets representing expression signatures of genetic and chemical perturbations.**

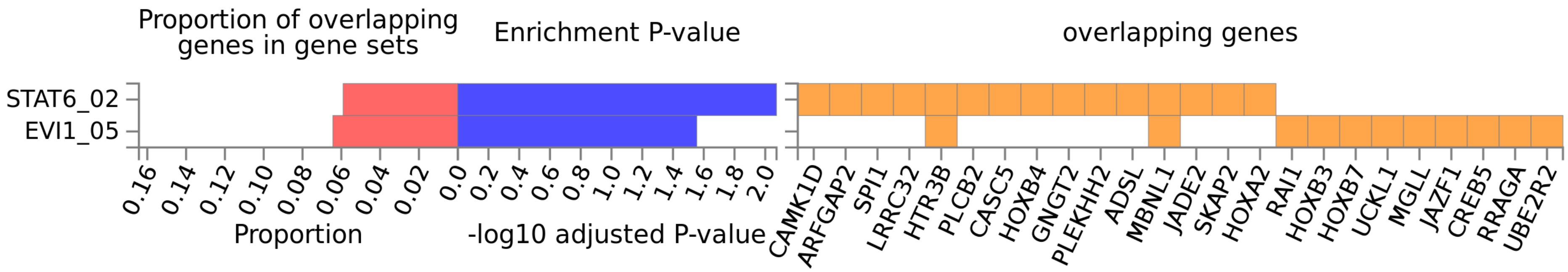

Fig A7: **Mapped genes (as done by** FUMA**) for the** 43 **pleiotropic loci detected by PLACO were tested for enrichment in MsigDB C3 gene sets that share upstream** ***cis*****-regulatory motifs which can function as potential transcription factor binding sites.**

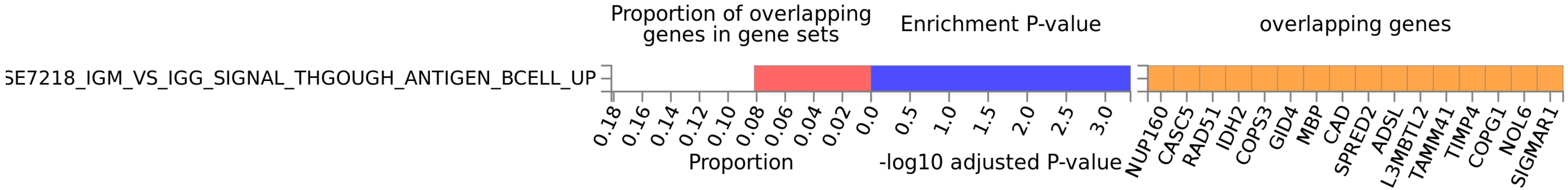

Fig A8: **Mapped genes (as done by** FUMA**) for the** 43 **pleiotropic loci detected by PLACO were tested for enrichment in MsigDB C3 gene sets representing cell states and perturbations within the immune system.**

References

[1] Huang, Y.-T. Genome-wide analyses of sparse mediation effects under composite null hypotheses. *Ann Appl Stat*, 13(1):60–84, 2019.

[2] Kim, J., Bai, Y., and Pan, W. An adaptive association test for multiple phenotypes with GWAS summary statistics. *Genet Epidemiol*, 39(8):651–663, 2015.

[3] Ray, D. and Boehnke, M. Methods for meta-analysis of multiple traits using GWAS summary statistics. *Genet Epidemiol*, 42(2):134–145, 2018.

[4] Lin, D.-Y. and Sullivan, P. Meta-analysis of genome-wide association studies with overlapping subjects. *Am J Hum Genet*, 85(6):862–872, 2009.

[5] Wang, T. and Elston, R. C. Improved power by use of a weighted score test for linkage disequilibrium mapping. *Am J Hum Genet*, 80(2):353–360, 2007.

[6] Basu, S. and Pan, W. Comparison of statistical tests for disease association with rare variants. *Genet Epidemiol*, 35(7):606–619, 2011.

[7] Ray, D., Li, X., Pan, W., Pankow, J. S., and Basu, S. A Bayesian partitioning model for the detection of multilocus effects in case-control studies. *Hum Hered*, 79(2):69–79, 2015.

[8] Zhang, Y., Qi, G., Park, J.-H., and Chatterjee, N. Estimation of complex effect-size distributions using summary-level statistics from genome-wide association studies across 32 complex traits. *Nat Genet*, 50(9):1318–1326, 2018.

[9] Liberzon, A., Subramanian, A., Pinchback, R., Thorvaldsdóttir, H., Tamayo, P., and Mesirov, J. P. Molecular signatures database (MSigDB) 3.0. *Bioinformatics*, 27(12):1739–1740, 2011.

[10] Kutmon, M., Riutta, A., Nunes, N., Hanspers, K., Willighagen, E. L., Bohler, A., Mélius, J., Waagmeester, A., Sinha, S. R., Miller, R., et al. WikiPathways: capturing the full diversity of pathway knowledge. *Nucleic Acids Res*, 44(D1):D488–D494, 2015.

[11] Meyer, T. E., Boerwinkle, E., Morrison, A. C., Volcik, K. A., Sanderson, M., Coker, A. L., Pankow, J. S., and Folsom, A. R. Diabetes genes and prostate cancer in the Atherosclerosis Risk in Communities study. *Cancer Epidemiol Biomarkers Prev*, 19(2):558–565, 2010.

[12] Mahajan, A., Taliun, D., Thurner, M., Robertson, N. R., Torres, J. M., Rayner, N. W., Payne, A. J., Steinthorsdottir, V., Scott, R. A., Grarup, N., et al. Fine-mapping type 2 diabetes loci to single-variant resolution using high-density imputation and islet-specific epigenome maps. *Nat Genet*, 50:1505–1513, 2018.

[13] Kircher, M., Witten, D. M., Jain, P., O'Roak, B. J., Cooper, G. M., and Shendure, J. A general framework for estimating the relative pathogenicity of human genetic variants. *Nat Genet*, 46(3):310–315, 2014.

[14] Boyle, A. P., Hong, E. L., Hariharan, M., Cheng, Y., Schaub, M. A., Kasowski, M., Karczewski, K. J., Park, J., Hitz, B. C., Weng, S., et al. Annotation of functional variation in personal genomes using RegulomeDB. *Genome Res*, 22(9):1790–1797, 2012.
