# Supplementary material for: A powerful method for pleiotropic analysis under composite null hypothesis identifies novel shared loci between Type 2 Diabetes and Prostate Cancer
Source: PLoS Genet. 2020 Dec 8;16(12):e1009218. doi: 10.1371/journal.pgen.1009218 (PMC7748289; doi:10.1371/journal.pgen.1009218)
Supplement: S1 Fig — Observed(−log10p-values) are plotted on the y-axis and Expected(−log10p-values) on the x-axis. Unequal study sample size, and equal case-control size assumed in each study. Study 1 has 4, 000 unrelated cases and 4, 000 unrelated controls. Study 2 has 1, 000 unrelated cases and 1, 000 unrelated controls, of which either 20%, 40%, 80% or 100% of the controls are shared between the two studies. Type I error performance of tests of pleiotropic effect of a genetic variant on the 2 traits is based on 9.99 million null variants with genetic effects that are either {β1 = 0 = β2} or {β1 = 0, β2 = log(1.15)} or {β1 = log(1.15), β2 = 0}. The gray shaded region represents a conservative 95% confidence interval for the expected distribution of p-values. (PDF) [file pgen.1009218.s002.pdf]

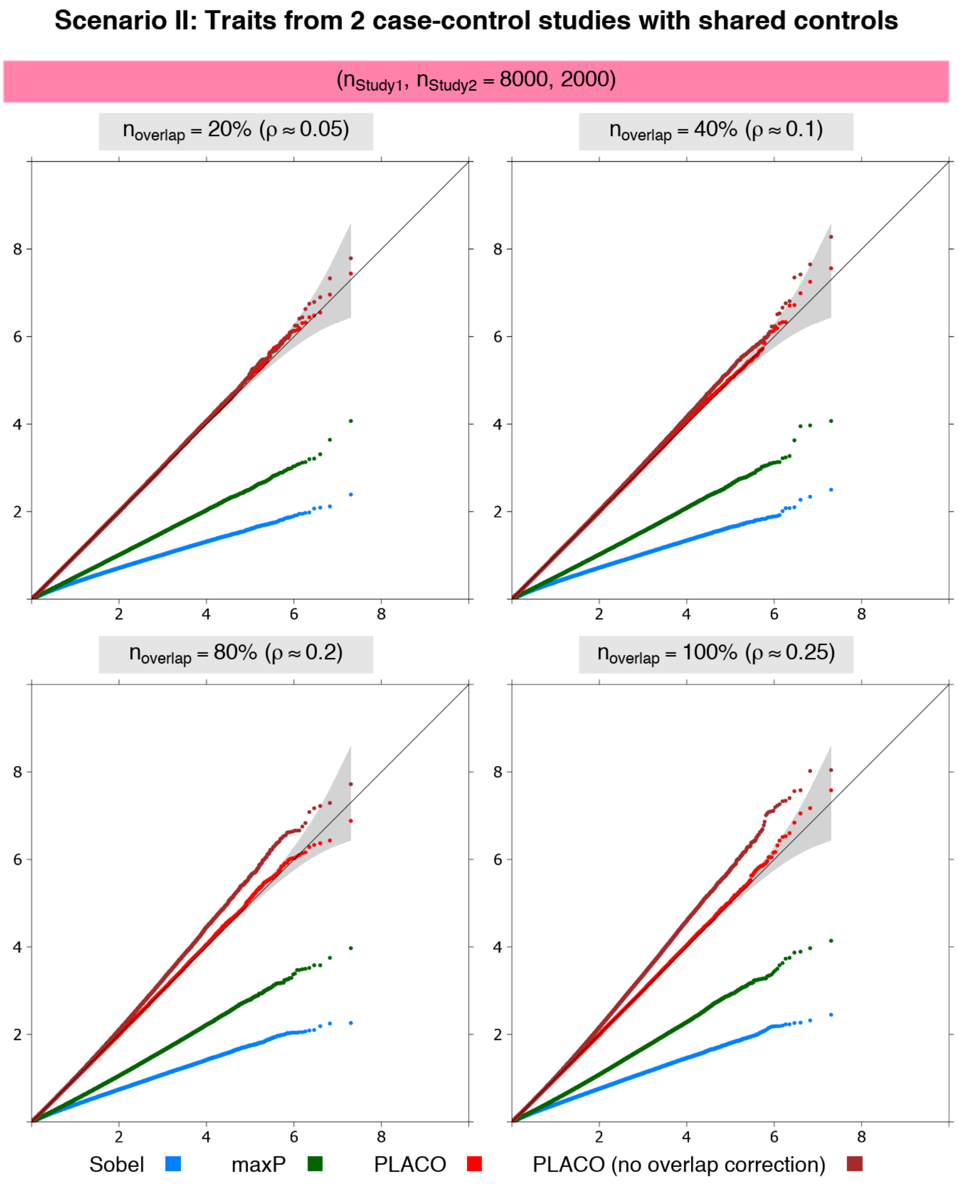

S1 Fig: Scenario II: QQ plots for the pleiotropic analysis of null data on traits from 2 case-control studies with different proportions of overlapping controls. Observed($-\log_{10}$p-values) are plotted on the y-axis and Expected($-\log_{10}$p-values) on the x-axis. Unequal study sample size, and equal case-control size assumed in each study. Study 1 has $4,000$ unrelated cases and $4,000$ unrelated controls. Study 2 has $1,000$ unrelated cases and $1,000$ unrelated controls, of which either 20%, 40%, 80% or 100% of the controls are shared between the two studies. Type I error performance of tests of pleiotropic effect of a genetic variant on the 2 traits is based on 9.99 million null variants with genetic effects that are either $\{\beta_1 = 0 = \beta_2\}$ or $\{\beta_1 = 0, \beta_2 = \log(1.15)\}$ or $\{\beta_1 = \log(1.15), \beta_2 = 0\}$. The gray shaded region represents a conservative 95% confidence interval for the expected distribution of p-values.
