# Supplementary material for: A powerful method for pleiotropic analysis under composite null hypothesis identifies novel shared loci between Type 2 Diabetes and Prostate Cancer
Source: PLoS Genet. 2020 Dec 8;16(12):e1009218. doi: 10.1371/journal.pgen.1009218 (PMC7748289; doi:10.1371/journal.pgen.1009218)
Supplement: S2 Fig — Observed(−log10p-values) are plotted on the y-axis and Expected(−log10p-values) on the x-axis. Type I error performance of tests of pleiotropic effect of a genetic variant on the 2 traits is based on 9.99 million null variants with genetic effects that are either {β1 = 0 = β2} or {β1 = 0, β2 explains 0.1% of Trait 2 variance} or {β1 explains 0.1% of Trait 1 variance, β2 = 0}. The gray shaded region represents a conservative 95% confidence interval for the expected distribution of p-values. P-values ≥10−12 are shown here. (PDF) [file pgen.1009218.s003.pdf]

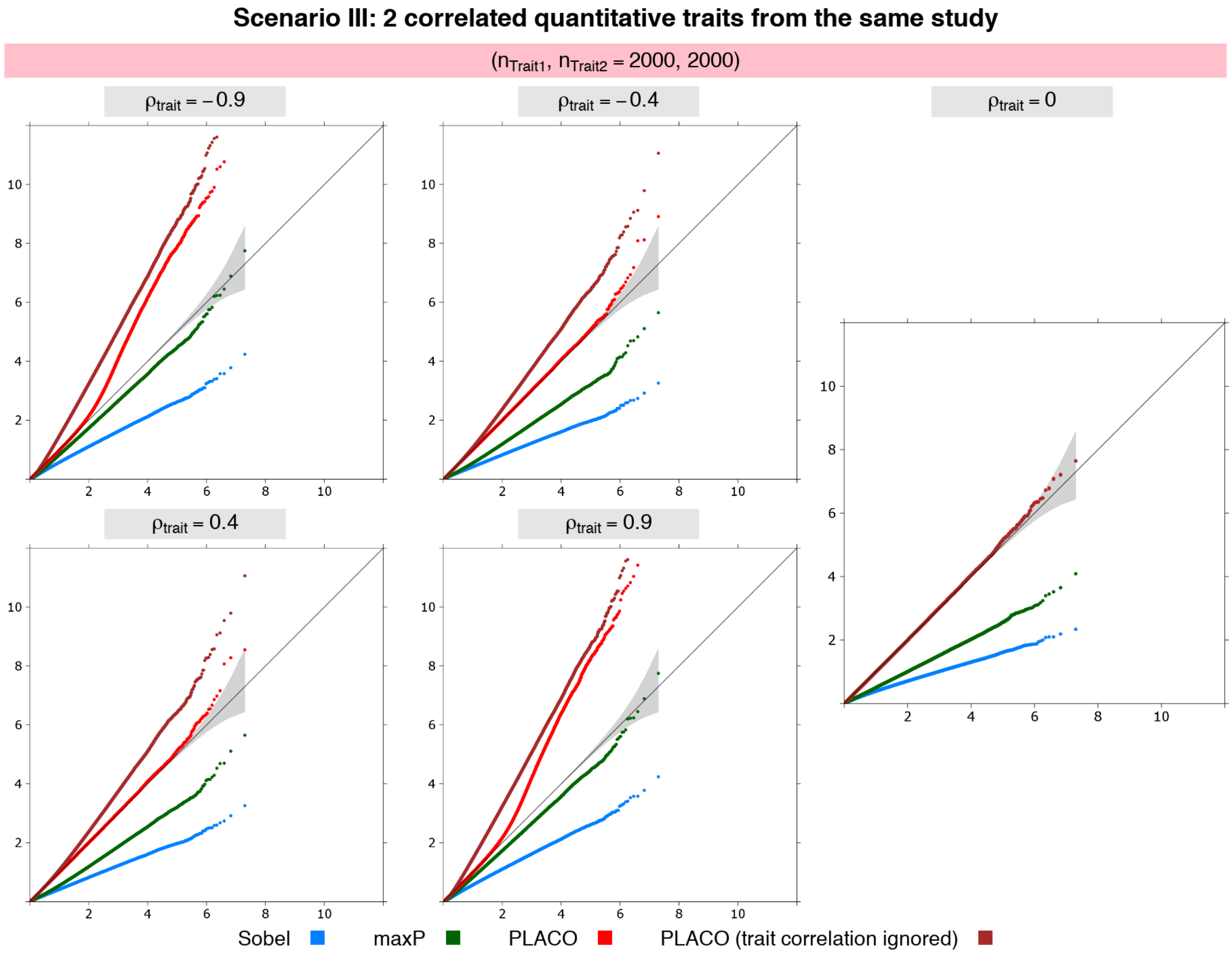

S2 Fig: Scenario III: QQ plots for the pleiotropic analysis of null data on 2 correlated traits where each trait is measured on the same 2,000 individuals. Observed($-\log_{10}$p-values) are plotted on the y-axis and Expected($-\log_{10}$p-values) on the x-axis. Type I error performance of tests of pleiotropic effect of a genetic variant on the 2 traits is based on 9.99 million null variants with genetic effects that are either $\{\beta_1 = 0 = \beta_2\}$ or $\{\beta_1 = 0, \beta_2$ explains 0.1% of Trait 2 variance$\}$ or $\{\beta_1$ explains 0.1% of Trait 1 variance, $\beta_2 = 0\}$. The gray shaded region represents a conservative 95% confidence interval for the expected distribution of p-values. P-values $\geq 10^{-12}$ are shown here.
