# Supplementary material for: A powerful method for pleiotropic analysis under composite null hypothesis identifies novel shared loci between Type 2 Diabetes and Prostate Cancer
Source: PLoS Genet. 2020 Dec 8;16(12):e1009218. doi: 10.1371/journal.pgen.1009218 (PMC7748289; doi:10.1371/journal.pgen.1009218)
Supplement: S4 Fig — (PDF) [file pgen.1009218.s005.pdf]

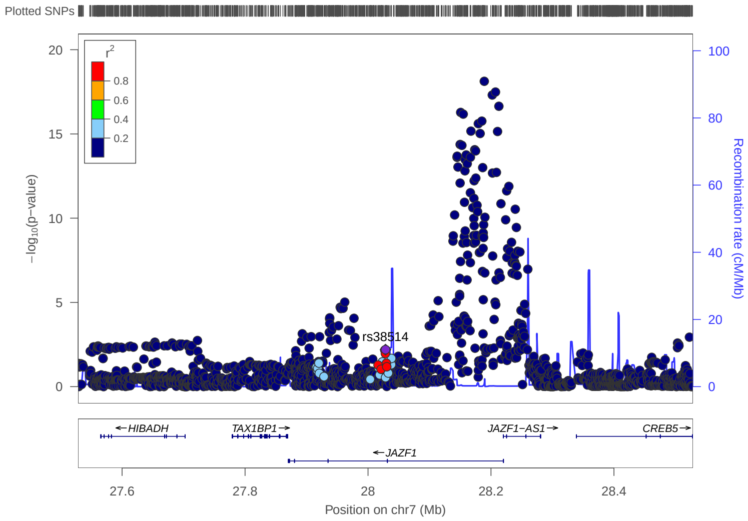

(a) T2D p-values

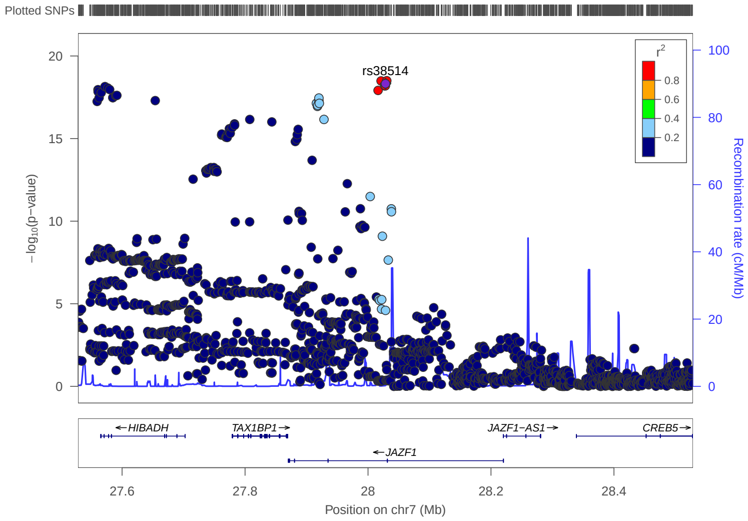

(b) PrCa p-values

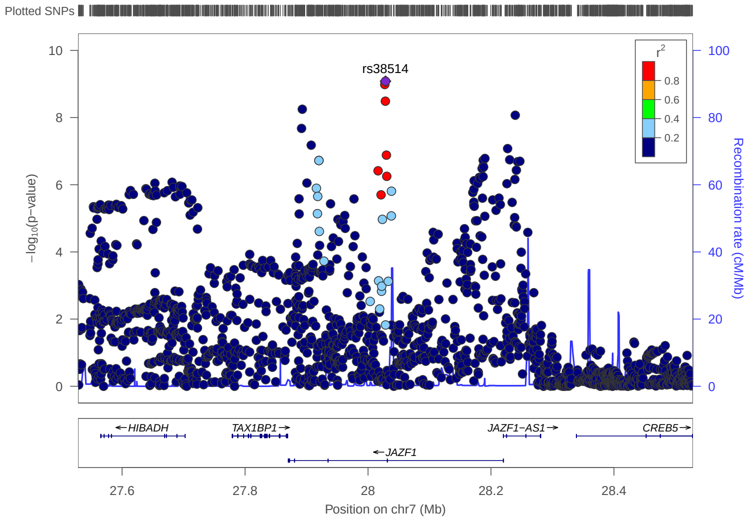

(c) PLACO p-values

S4 Fig: Locuszoom plots of association p-values for variants in and around gene *JAZF1*.
