# Supplementary material for: A powerful method for pleiotropic analysis under composite null hypothesis identifies novel shared loci between Type 2 Diabetes and Prostate Cancer
Source: PLoS Genet. 2020 Dec 8;16(12):e1009218. doi: 10.1371/journal.pgen.1009218 (PMC7748289; doi:10.1371/journal.pgen.1009218)
Supplement: S9 Fig — (PDF) [file pgen.1009218.s010.pdf]

Ray, D. and Chatterjee, N. *A powerful method for pleiotropic analysis under composite null hypothesis identifies novel shared loci between type 2 diabetes and prostate cancer.* PLoS Genet.; DOI: 10.1371/journal.pgen.1009218

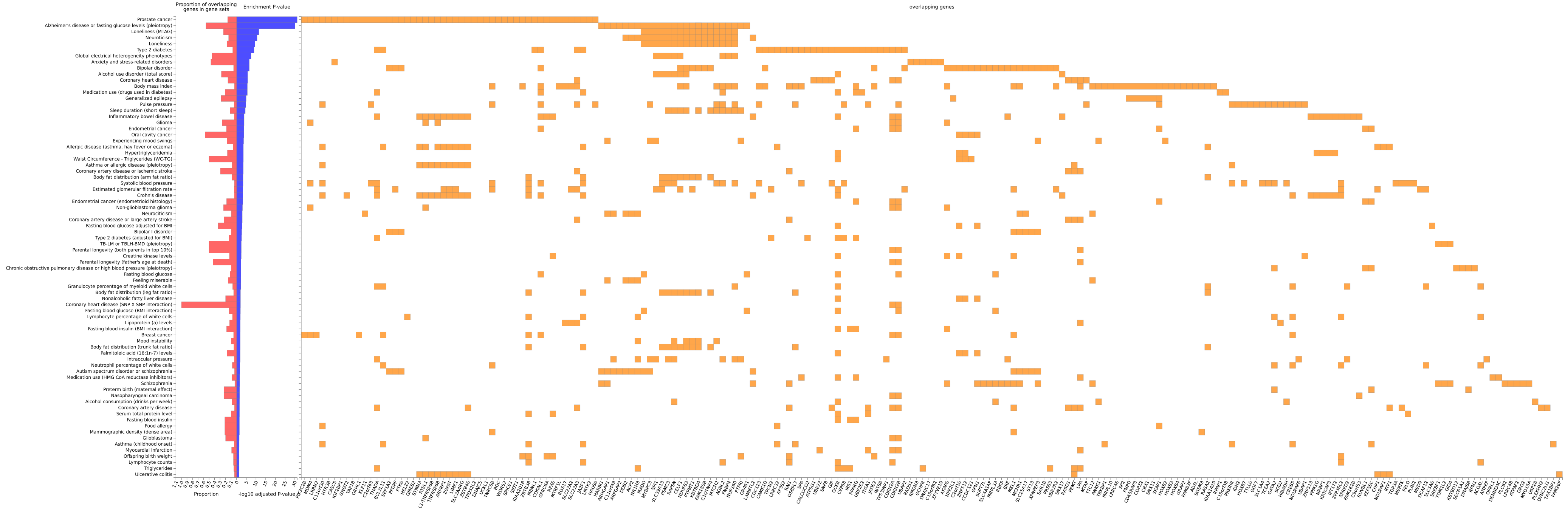

S9 Fig: Mapped genes (as done by FUMA) for the 43 pleiotropic loci detected by PLACO were tested for enrichment in GWAS catalog reported genes across diseases and traits.
