# Supplementary material for: A powerful method for pleiotropic analysis under composite null hypothesis identifies novel shared loci between Type 2 Diabetes and Prostate Cancer
Source: PLoS Genet. 2020 Dec 8;16(12):e1009218. doi: 10.1371/journal.pgen.1009218 (PMC7748289; doi:10.1371/journal.pgen.1009218)
Supplement: S10 Fig — (PDF) [file pgen.1009218.s011.pdf]

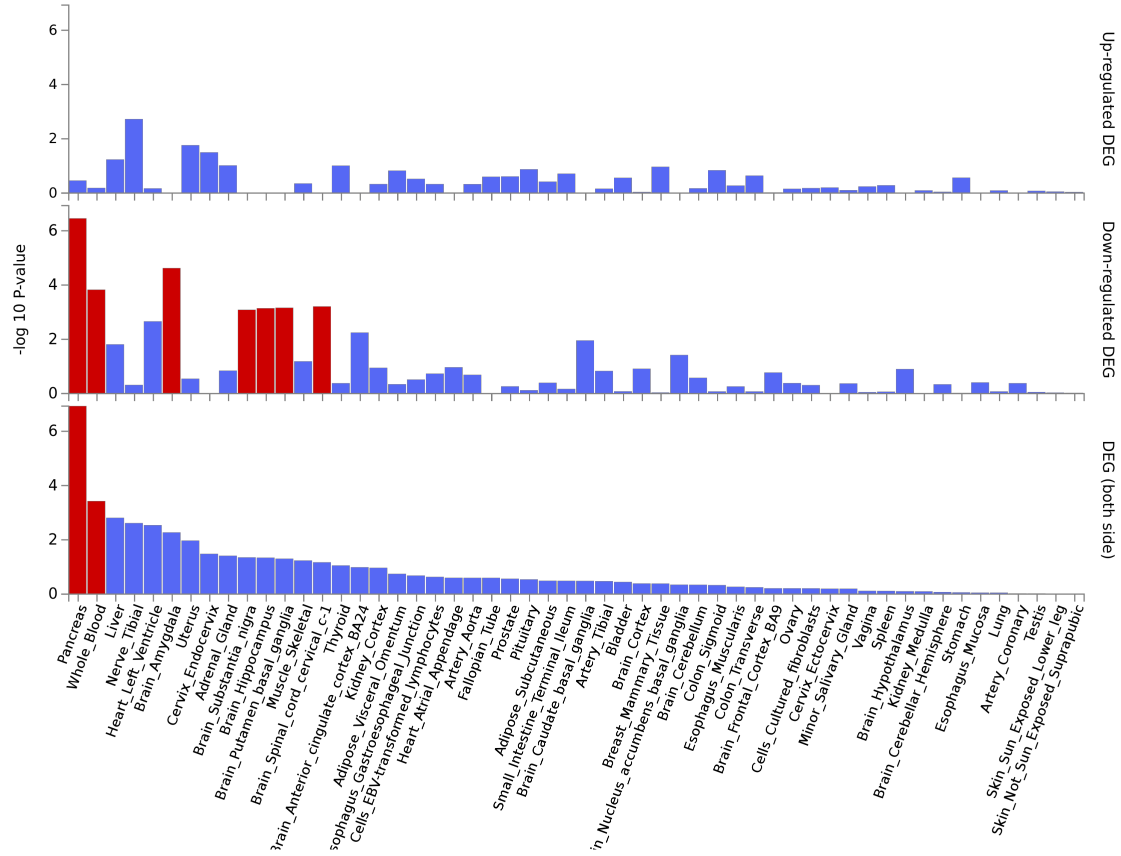

S10 Fig: Mapped genes (as done by `FUMA`) for the 43 pleiotropic loci detected by PLACO were tested against each of the Differentially Expressed Gene (DEG) sets pre-calculated from GTEx v8 tissue data from 53 tissue types.
