# Supplementary material for: A powerful method for pleiotropic analysis under composite null hypothesis identifies novel shared loci between Type 2 Diabetes and Prostate Cancer
Source: PLoS Genet. 2020 Dec 8;16(12):e1009218. doi: 10.1371/journal.pgen.1009218 (PMC7748289; doi:10.1371/journal.pgen.1009218)
Supplement: S13 Fig — Tissues considered are whole blood from eQTLGen Consortium; and adipose, liver, muscle-skeletal, pancreas, and prostate tissues from GTEx v8. (PDF) [file pgen.1009218.s014.pdf]

Ray, D. and Chatterjee, N. *A powerful method for pleiotropic analysis under composite null hypothesis identifies novel shared loci between type 2 diabetes and prostate cancer.* PLoS Genet.; DOI: 10.1371/journal.pgen.1009218

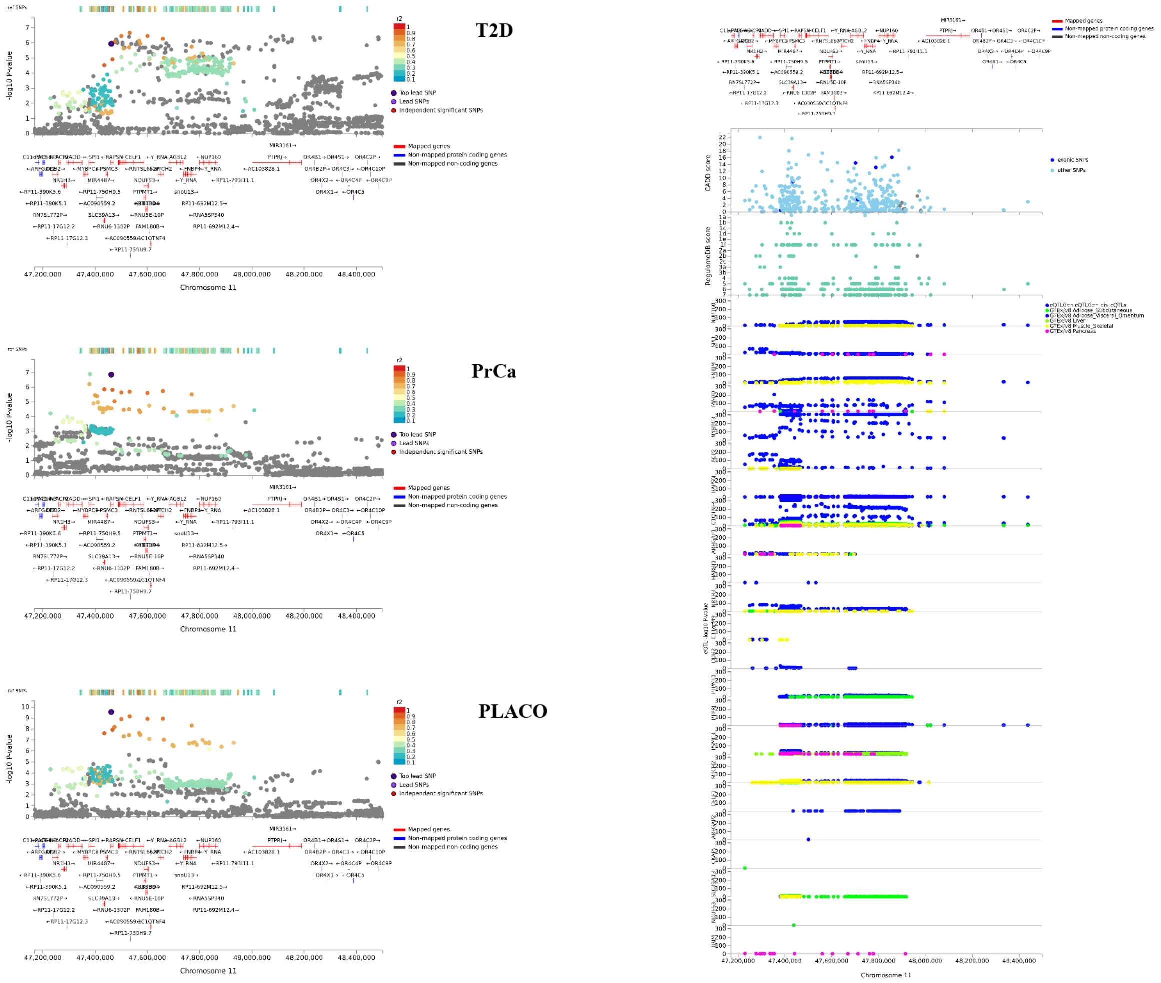

S13 Fig: Regional association plot of significant pleiotropic locus near *RAPSN* with annotations such as CADD scores, RegulomeDB scores, and *cis* eQTL association p-values from 6 tissues. Tissues considered are whole blood from eQTLGen Consortium; and adipose, liver, muscle-skeletal, pancreas, and prostate tissues from GTEx v8.
