# Supplementary material for: A powerful method for pleiotropic analysis under composite null hypothesis identifies novel shared loci between Type 2 Diabetes and Prostate Cancer
Source: PLoS Genet. 2020 Dec 8;16(12):e1009218. doi: 10.1371/journal.pgen.1009218 (PMC7748289; doi:10.1371/journal.pgen.1009218)
Supplement: S1 Table — Each study has 9.9 × 10^6^ null variants (i.e., variants under H00 or H01 or H02) and m non-null (pleiotropic) variants, where m takes values 0, 100, 300, 500, 1000, 3000, 5000 or 10000. Five different choices of odds ratios of association of m non-null variants with Traits 1 and 2 are considered. The total number of true positives (non-null variants) detected (#TP) and the total number of false positives detected (#FP) are reported. PLACO’s performance for both genome-wide threshold 5 × 10^−8^ (or equivalently family-wise error rate (FWER) of 5%) and global false discovery rate (FDR) of 5% are reported, while GPA’s performance is based on global FDR of 5%. (PDF) [file pgen.1009218.s018.pdf]

S1 Table: Scenario I: Comparison of PLACO and GPA in terms of error control and power for 2 independent case-control studies, where each study has 1,000 unrelated cases and 1,000 unrelated controls. Each study has 9.9×10^6 null variants (i.e., variants under H_{00} or H_{01} or H_{02}) and m non-null (pleiotropic) variants, where m takes values 0, 100, 300, 500, 1000, 3000, 5000 or 10000. Five different choices of odds ratios of association of m non-null variants with Traits 1 and 2 are considered. The total number of true positives (non-null variants) detected (#TP) and the total number of false positives detected (#FP) are reported. PLACO's performance for both genome-wide threshold 5×10^{-8} (or equivalently family-wise error rate (FWER) of 5%) and global false discovery rate (FDR) of 5% are reported, while GPA's performance is based on global FDR of 5%.

Non-null OR	Simulation type		PLACO				GPA	
	# null SNPs	# non-null SNPs	FWER 5%		FDR 5%		FDR 5%	
			#TP	#FP	#TP	#FP	#TP	#FP
**(1.5, 1.5)**	9.9×10^6	0	0	1	0	0	0	0
	9.9×10^6	100	4	1	2	1	0	0
	9.9×10^6	300	20	1	29	2	0	0
	9.9×10^6	500	35	1	66	2	0	0
	9.9×10^6	1000	67	1	161	4	0	0
	9.9×10^6	3000	189	1	773	34	664	25
	9.9×10^6	5000	340	1	1544	74	1370	62
	9.9×10^6	10^4	708	1	3879	175	3584	154
**(1.7, 1.7)**	9.9×10^6	0	0	1	0	0	0	0
	9.9×10^6	100	44	1	61	2	0	0
	9.9×10^6	300	147	1	209	8	0	0
	9.9×10^6	500	238	1	354	13	0	0
	9.9×10^6	1000	465	1	770	33	764	41
	9.9×10^6	3000	1390	1	2497	112	2520	177
	9.9×10^6	5000	2318	1	4329	198	4393	307
	9.9×10^6	10^4	4736	1	8980	432	9142	685
**(1.5, 1/1.5)**	9.9×10^6	0	0	1	0	0	0	0
	9.9×10^6	100	3	1	3	1	0	0
	9.9×10^6	300	10	1	12	1	0	0
	9.9×10^6	500	19	1	28	2	0	0
	9.9×10^6	1000	39	1	85	2	0	0
	9.9×10^6	3000	133	1	478	20	428	12
	9.9×10^6	5000	236	1	977	41	876	36
	9.9×10^6	10^4	476	1	2609	127	2321	100
**(1.7, 1/1.7)**	9.9×10^6	0	0	1	0	0	0	0
	9.9×10^6	100	19	1	21	2	0	0
	9.9×10^6	300	64	1	115	2	0	0
	9.9×10^6	500	122	1	243	9	0	0
	9.9×10^6	1000	257	1	577	26	552	22
	9.9×10^6	3000	831	1	2035	94	2040	101
	9.9×10^6	5000	1380	1	3576	157	3608	207
	9.9×10^6	10^4	2813	1	7726	377	7896	464
**(1.7, 1.2)**	9.9×10^6	0	0	1	0	0	0	0
	9.9×10^6	100	0	1	0	0	0	0
	9.9×10^6	300	4	1	0	0	0	0
	9.9×10^6	500	9	1	10	1	0	0
	9.9×10^6	1000	17	1	23	2	118	3
	9.9×10^6	3000	40	1	109	2	707	29
	9.9×10^6	5000	78	1	267	9	1442	61
	9.9×10^6	10^4	144	1	844	35	3607	155

# denotes total number (e.g. #TP denotes total number of true positives detected).
