# Supplementary material for: A powerful method for pleiotropic analysis under composite null hypothesis identifies novel shared loci between Type 2 Diabetes and Prostate Cancer
Source: PLoS Genet. 2020 Dec 8;16(12):e1009218. doi: 10.1371/journal.pgen.1009218 (PMC7748289; doi:10.1371/journal.pgen.1009218)
Supplement: S2 Table — Each study has 9.9 × 10^6^ null variants (i.e., variants under H00 or H01 or H02) and m non-null (pleiotropic) variants, where m takes values 0, 100, 300, 500, 1000, 3000, 5000 or 10000. Five different choices of odds ratios of association of m non-null variants with Traits 1 and 2 are considered. The total number of true positives (non-null variants) detected (#TP) and the total number of false positives detected (#FP) are reported. PLACO’s performance for both genome-wide threshold 5 × 10^−8^ (or equivalently family-wise error rate (FWER) of 5%) and global false discovery rate (FDR) of 5% are reported, while GPA’s performance is based on global FDR of 5%. (PDF) [file pgen.1009218.s019.pdf]

**S2 Table: Scenario I: Comparison of PLACO and GPA in terms of error control and power for 2 independent case-control studies, where Study 1 has 4 times sample size as Study 2, and Study 2 has $1,000$ unrelated cases and $1,000$ unrelated controls.** Each study has $9.9 \times 10^6$ null variants (i.e., variants under $H_{00}$ or $H_{01}$ or $H_{02}$) and $m$ non-null (pleiotropic) variants, where $m$ takes values 0, 100, 300, 500, 1000, 3000, 5000 or 10000. Five different choices of odds ratios of association of $m$ non-null variants with Traits 1 and 2 are considered. The total number of true positives (non-null variants) detected (#TP) and the total number of false positives detected (#FP) are reported. PLACO's performance for both genome-wide threshold $5 \times 10^{-8}$ (or equivalently family-wise error rate (FWER) of 5%) and global false discovery rate (FDR) of 5% are reported, while GPA's performance is based on global FDR of 5%.

| OR | Simulation type | | PLACO | | | | GPA | |
|---|---|---|---|---|---|---|---|---|
| | # null SNPs | # non-null SNPs | FWER 5% | | FDR 5% | | FDR 5% | |
| | | | #TP | #FP | #TP | #FP | #TP | #FP |
| **(1.5, 1.5)** | $9.9 \times 10^6$ | 0 | 0 | 0 | 0 | 0 | 0 | 0 |
| | $9.9 \times 10^6$ | 100 | 60 | 0 | 68 | 5 | 1 | 0 |
| | $9.9 \times 10^6$ | 300 | 185 | 0 | 239 | 19 | 163 | 2 |
| | $9.9 \times 10^6$ | 500 | 310 | 0 | 408 | 30 | 471 | 66 |
| | $9.9 \times 10^6$ | 1000 | 611 | 0 | 859 | 56 | 981 | 287 |
| | $9.9 \times 10^6$ | 3000 | 1807 | 0 | 2645 | 142 | 2968 | 584 |
| | $9.9 \times 10^6$ | 5000 | 3024 | 0 | 4518 | 224 | 4964 | 882 |
| | $9.9 \times 10^6$ | $10^4$ | 6004 | 0 | 9164 | 456 | 9942 | 1595 |
| **(1.7, 1.7)** | $9.9 \times 10^6$ | 0 | 0 | 0 | 0 | 0 | 0 | 0 |
| | $9.9 \times 10^6$ | 100 | 94 | 0 | 99 | 8 | 76 | 0 |
| | $9.9 \times 10^6$ | 300 | 284 | 0 | 298 | 22 | 300 | 76 |
| | $9.9 \times 10^6$ | 500 | 472 | 0 | 497 | 34 | 500 | 107 |
| | $9.9 \times 10^6$ | 1000 | 961 | 0 | 996 | 63 | 1000 | 182 |
| | $9.9 \times 10^6$ | 3000 | 2906 | 0 | 2991 | 156 | 3000 | 440 |
| | $9.9 \times 10^6$ | 5000 | 4854 | 0 | 4985 | 247 | 5000 | 676 |
| | $9.9 \times 10^6$ | $10^4$ | 9711 | 0 | 9974 | 499 | 10000 | 1238 |
| **(1.5, 1/1.5)** | $9.9 \times 10^6$ | 0 | 0 | 0 | 0 | 0 | 0 | 0 |
| | $9.9 \times 10^6$ | 100 | 51 | 0 | 59 | 4 | 0 | 0 |
| | $9.9 \times 10^6$ | 300 | 146 | 0 | 189 | 16 | 286 | 124 |
| | $9.9 \times 10^6$ | 500 | 243 | 0 | 337 | 27 | 486 | 258 |
| | $9.9 \times 10^6$ | 1000 | 493 | 0 | 737 | 54 | 973 | 309 |
| | $9.9 \times 10^6$ | 3000 | 1385 | 0 | 2331 | 126 | 2947 | 610 |
| | $9.9 \times 10^6$ | 5000 | 2294 | 0 | 4043 | 206 | 4932 | 906 |
| | $9.9 \times 10^6$ | $10^4$ | 4571 | 0 | 8416 | 422 | 9916 | 1627 |
| **(1.7, 1/1.7)** | $9.9 \times 10^6$ | 0 | 0 | 0 | 0 | 0 | 0 | 0 |
| | $9.9 \times 10^6$ | 100 | 87 | 0 | 92 | 6 | 98 | 5 |
| | $9.9 \times 10^6$ | 300 | 264 | 0 | 284 | 21 | 300 | 90 |
| | $9.9 \times 10^6$ | 500 | 444 | 0 | 480 | 32 | 500 | 121 |
| | $9.9 \times 10^6$ | 1000 | 890 | 0 | 970 | 61 | 1000 | 202 |
| | $9.9 \times 10^6$ | 3000 | 2672 | 0 | 2936 | 155 | 3000 | 479 |
| | $9.9 \times 10^6$ | 5000 | 4466 | 0 | 4908 | 243 | 5000 | 731 |
| | $9.9 \times 10^6$ | $10^4$ | 8929 | 0 | 9858 | 495 | 10000 | 1329 |
| **(1.7, 1.2)** | $9.9 \times 10^6$ | 0 | 0 | 0 | 0 | 0 | 0 | 0 |
| | $9.9 \times 10^6$ | 100 | 24 | 0 | 27 | 2 | 0 | 0 |
| | $9.9 \times 10^6$ | 300 | 64 | 0 | 90 | 5 | 300 | 175 |
| | $9.9 \times 10^6$ | 500 | 112 | 0 | 171 | 15 | 500 | 189 |
| | $9.9 \times 10^6$ | 1000 | 218 | 0 | 377 | 29 | 1000 | 277 |
| | $9.9 \times 10^6$ | 3000 | 688 | 0 | 1348 | 79 | 3000 | 606 |
| | $9.9 \times 10^6$ | 5000 | 1120 | 0 | 2414 | 128 | 5000 | 906 |
| | $9.9 \times 10^6$ | $10^4$ | 2317 | 0 | 5276 | 257 | 10000 | 1606 |

# denotes total number (e.g. #TP denotes total number of true positives detected).
